# The Interplay Between Use of Biological Therapies, Psychological State, and the Microbiome in IBD

**DOI:** 10.3389/fmed.2022.788992

**Published:** 2022-07-19

**Authors:** Paris Tavakoli, Ute Vollmer-Conna, Dusan Hadzi-Pavlovic, Xabier Vázquez-Campos, Michael Carl Grimm

**Affiliations:** ^1^St. George and Sutherland Clinical School, University of New South Wales, Sydney, NSW, Australia; ^2^School of Psychiatry, Faculty of Medicine, University of New South Wales, Sydney, NSW, Australia; ^3^School of Biotechnology and Biomolecular Sciences, Faculty of Science, University of New South Wales, Sydney, NSW, Australia

**Keywords:** Crohn's disease, ulcerative colitis, inflammatory bowel disease, gut microbiome, psychological state

## Abstract

**Background:**

This study examines longitudinal bio-psychological dynamics and their interplay in IBD patients undergoing conventional and biological therapies.

**Methods:**

Fifty IBD participants (24 UC, 26 CD) in clinical remission were followed for 12 months. Complete longitudinal datasets, biological samples, validated scores of psychological status were collected monthly for analysis of association. Microbiome analysis was performed to identify microbial dynamics and signatures. Patients were grouped on disease phenotype (CD, UC) and mode of treatment (biological therapies, non-biological treatment). General linear models, mixed models, cluster analysis, and analyses of variance were used to examine the longitudinal trends of the variables and their associations over time. Results were corrected for multiple testing.

**Results:**

Results substantiated different interactions between biological therapy and longitudinal trends of inflammatory biomarkers in remission CD and UC patients as well as significant differences between CD and UC patients in their psychological measures during clinical remission, with UC patients having inferior condition compared to CD. A significant reduction in microbial diversity in CD patients compared to UC was identified. Results characterized considerable differences in longitudinal microbial profile between those taking and not taking biological treatment in UC patients, but not in CD patients.

**Conclusion:**

A different trajectory of interdependence was identified between psychological state, sleep, and microbial dynamics with mode of treatment when compared between CD and UC patients. Further studies should investigate the causal relationships between bio-psychological factors for improved treatment purposes.

## Introduction

Study of chronic inflammatory diseases such as inflammatory bowel diseases (IBDs), generates numerous challenges including assessing inflammatory pathways that might be common between different chronic inflammatory disorders with either shared or disease-specific mechanisms. One approach is to examine the relationship between major contributing factors over time to identify key drivers and their interplay. Several chronic conditions including IBDs have arisen and increased in incidence during the past century points to powerful environmental influences, perhaps as a product of industrialization and modernization (1, 2). The more recent exploration of IBD genetics has shown genetic variances selectively associated with IBD (3, 4), but also made clear that no single or combination of genetic variation can fully explain Crohn's disease (CD) or ulcerative colitis (UC) (5, 6). Since the early study of the IBDs, immunological mechanisms were the dominant area of research. Further, microbiome and genetic studies indeed supported the role of immune cells (mainly type 1, type 2 and type 17 T cells) and cytokines (7). These findings were incentives to tackle and block principal mediators in immune responses with the therapeutic aim to control inflammation and potentially alter the natural history of disease. It has long been proposed that gut bacteria play an important role in the pathogenesis of IBD through their direct interaction with the intestinal mucosa. IBDs are characterized by immune dysregulation in genetically susceptible patients and it seems that gut microbiota are the target of this inapt immune response either due to loss of tolerance toward commensal bacteria or secondary to an altered microbial diversity and/or function (8, 9). Many subsequent studies present convincing evidence confirming the involvement of the enteric bacteria in pathogenesis of IBD. A range of bacteria is stated to have aggressive or protective functions in intestinal inflammatory disorders such as Crohn's disease; for example, phlogistic effects of adherent-invasive *Escherichia coli* (10) and protective effects of *Faecalibacterium prausnitzii* (11). To examine the integrated impact of gut microbiota in the pathogenesis of IBDs, it is important to incorporate microbiome data with other data related to immune modulation, genetics, psychological and physiological factors.

IBDs are chronic debilitating disorders which may affect many aspects of the sufferer's life. They can add to the psychological burden including high levels of perceived stress (12), negative mood and depression (13), and anxiety compared to the healthy population (14). The prevalence estimate of both depression and anxiety were higher in IBD patients—even among patients in remission—than in the general population (15–17).

These many factors suggest that disease mechanisms in IBDs are multifaceted and gut inflammation is the product of complex pathways in addition to known immune response types, notwithstanding the direction of newer targeted therapies (6, 18). Longitudinal assessments of biological and psychological factors and understanding their temporal trajectories in the course of the disease are essential to clarifying vulnerabilities and individual differences in IBD patients. Previous published studies were limited in the number of risk factors examined or they lacked time series analysis of such disease contributing factors (17, 19–21), therefore this study has been designed to examine how multiple contributing factors and their interrelationships have influenced the disease's course over time and how different interactions are represented in IBD patients who received biological treatment compared to those on conventional treatment.

## Methods

### Cohort Demographics

Patients with confirmed diagnoses of IBD who met all the inclusion criteria and none of the exclusion criteria (see Supplementary Information) were recruited from gastroenterology departments, IBD clinics and endoscopy units based at two tertiary referral hospitals in Sydney, Australia, between Oct 2015 and August 2017. Study participants were in complete clinical remission based on their disease activity indices; partial Mayo score for UC <2 (22), Crohn's disease activity index (CDAI) for CD <150 (23) and/or Harvey Bradshaw Index for CD <5 (23–25) confirmed by their gastroenterologists, and supported by endoscopic and histological results, if available. Baseline data were collected, and longitudinal data accumulated monthly. Data comprised scores related to symptoms of psychological state including perceived stress (PSQ) (26, 27), depression- anxiety and stress (DASS) (28, 29), depression in medically ill (DMI) (30, 31), personality characteristics, i.e., negative affectivity (NA) and social inhibition (SI) traits (32), wellbeing scores (33–35) and sleep quality (PSQI) (36) with clinical course and disease activity as a measure of outcome. To build the outcome, all variables including severe disease symptom/s and flare events were considered through formalized follow up assessments by study investigators. We studied the longitudinal dynamics of multiple contributing factors to disease activity from a cohort of 50 IBD patients (CD, *n* = 26; UC, *n* = 24; Tables 1, 2). IBD participants were grouped in disease types (CD or UC) and subdivided based on use or not of biological treatments. Monthly blood and stool samples were collected for assessment of serum C-reactive protein (CRP), fecal calprotectin (FC) levels and microbiome dynamics. The microbiome composition in each sample was determined by sequencing the V4 region of the 16S rRNA gene and a total of 3.3 million contigs were retained after quality control and subsampling. To determine the links between the gut microbiome and clinical components, we collected clinical data including inflammatory biomarkers, clinical indices for disease activity, and self-administered validated questionnaires to quantify psychological state on a monthly basis for a period of 12 months while participants still met the inclusion criteria for the study. Data related to routine medications, use of antibiotics and probiotics and disease activity scores were registered at monthly follow up visits. The end point was at 12 months follow-up or at confirmation of the onset of relapse; in the latter case the last assessment reflected the state at the time of disease relapse. The study was approved by the Human Research Ethics Committee of South Eastern Sydney Local Health District (Ref: 15/094 HREC/15/POWH 245−20 Aug).

**Table 1:** Participants' distribution in each group of diagnosis and treatment options.

**IBD phenotypes**	***F***	***M***	**On-biologic therapy**	**Non-biologic therapy**	**Total**
CD	12	14	10	16	26
UC	9	15	5	19	24
Total	21	29	15	35	**50**

*An IBD cohort of 50 (CD, n = 26; UC, n = 24) were enrolled and followed up for the period of 12 months. One UC participant withdrew after 2 months of follow up*.

**Table 2:** Sample distribution in IBD groups.

**IBD phenotypes**	**Number of** **samples—Blood**	**Number of** **serum** **samples/on-** **biologic** **therapy**	**Number of** **serum** **samples/non-** **biologic** **therapy**	**Number of** **samples—Stool**	**Number of stool** **samples** **included in** **microbial** **analysis**	**Number of stool** **samples/on-** **biologic** **therapy**	**Number of stool** **samples/non-** **biologic** **therapy**
CD	273	104^*^	169	273	98^**^	30^*^	68
UC	235	53	182	235	85^***^	19	66
Total	508	157	351	508	183^****^	49	134

*One hundred eighty-eight samples from 50 IBD patients were included in the analysis. An additional 5 samples were removed from calculation at the sub-sampling level after sequencing and filtering as they did not have a minimum of 10,000 sequences.*

* *One CD participant was on biologic on 1st month and on non-biologic therapy for the rest of assessment period, so added to 508 total samples in both groups of treatment for rest of assessment period. Samples from this participant were 1 (bio) + 10 (non-bio).*

** *3 CD stool samples did not qualify for microbiome results and were excluded (only 98 stool samples were included in the results therefore the numbers are 98 instead of 101 in this group).*

*** *2 UC stool samples did not qualify for microbiome results and were excluded (only 85 stool samples were included in the results instead of 87).*

**** *5 stool samples (3 CD and 2 UC) were removed as non-qualified therefore the total was 183 (49 + 134) instead of 188*.

### Sample Collection

Fecal samples were self-collected by participants and were aliquoted and stored at −80°C together with original collection pots for DNA extraction and quantifying of FC. The concentration of FC was assessed by commercially available enzyme-linked immunosorbent assay (ELISA; Calprotectin Elisa Buhlmann Laboratories, S100A8 and S100A9), according to the manufacturer's protocol. Twenty ml peripheral blood samples were drawn and centrifuged (at 2,000 g for 15 min). Serum samples were aliquoted and stored at −80°C to be used for CRP quantitation by high sensitivity ELISA (37). For microbiome assessment, stool samples were taken from collections at months 1, 4, 8, and 12; where there were any missing samples, the sample from the preceding available month was used.

### DNA Extraction and Amplification

Genomic DNA was extracted from 0.11 to 0.12 g of fecal material of each sample using the Allprep Power Viral DNA/RNA Kit (Qiagen) DNA extraction protocol. Briefly, the concentration of the extracted DNAs was quantified by Nanodrop mass spectrophotometry (38) and Qubit 2.0 fluorometer (Invitrogen) according to the manufacturer's instructions before dilution to 20 ng/μl. For DNA concentrations of <5 ng/μl (39 extractions), SYBR-Green-based qPCR assay was performed to quantify the absolute amount of a target sequence, to compare relative amounts of a target sequence between samples and to analyse whether they were amplifiable (Supplementary Figure 1; Supplementary Table 1 qPCR plot and table). DNA was submitted for sequencing at the Ramaciotti Center for Genomics (Australia).

### Library Preparation

Barcoding PCR for bacterial and archaeal 16S rRNA genes was carried out using a mix of 10 μL of HotMasterMix (5 PRIME), 0.2 μM of each primer and 1 μL of DNA template. Barcoded PCR primers based on 515F, and 806R (39). Reactions were kept at 94°C for 3 min for denaturing, followed by 35 cycles of denaturation at 94°C for 45 s, annealing at 50°C for 1 min and elongation at 72°C for 1 min 30s, ending with a final elongation at 72°C for 10 min and final hold at 4°C. All PCRs were carried out in 25 μL volumes. PCR concentrations were normalized and pooled using SequalPrep^™^ Normalization Plate Kit (ThermoFisher) according to the manufacturer's instructions. The library was purified using Axygen^®^ AxyPrep^™^ Mag PCR Clean-Up Kit (Fisher Biotec) as per the manufacturer's instructions. Concentration and quality of the pooled library were checked with Qubit^®^ and the library size on an Agilent 2,200 TapeStation instrument.

### Community Analysis

Raw sequencing data were processed with the OTUreporter pipeline (40), based on mothur v1.39.5 (41) and according to the MiSeq SOP. Samples with a length between 228 and 278 bp were retained and those with homopolymers longer than 8 bp were removed. Sequences were grouped into OTUs based on 97% similarity using the OptiClust algorithm. From each patient, quarterly microbiome samples with matched FC concentrations were sub-selected for use in downstream analysis.

### Statistical Analysis

Following the power analysis estimation (80%) to detect significant (two sided *p*-values ≤ 0.05) correlations (42) cluster analysis was used to examine whether categories of respondents (IBD patients) share common characteristics within clusters at the baseline and maintain the same properties over time (43). Utilizing SPSS 25 (SPSS Inc), general linear models, non-parametric methods, time series analysis and mixed model analysis were used to examine longitudinal data related to psychological and biological measures, their linear/quadratic trends and differences between two groups of treatment options over time (44). Furthermore, a regression analysis using panel data was conducted applying Stata (Stata V16; Stata Statistical Software: Release 16. College Station, TX: StataCorp LLC. 2019) to apply corrections for multiple analysis. Microbial profile analysis was carried out using the phyloseq (45) and microbiome (https://github.com/microbiome/microbiome) R packages to import and graph data, and vegan was used to perform differential abundance testing (46–48). Wilcoxon signed-rank test, and Permutational multivariate analysis of variance (PERMANOVA) test, were applied to examine statistical significance of differences in some bacteria abundances at family level and to evaluate compositional differences between the CD and UC groups, and within treatment modalities (49, 50). Bray Curtis dissimilarity matrices were used as input for PERMANOVA to evaluate compositional differences between the CD and UC and treatment modalities. Analysis of Variance (ANOVA) was used onto beta dispersion test output to validate significant result obtained by PERMANOVA. Community and species diversity were estimated using the Shannon diversity index (51) while species richness estimates were generated using Chao1 (52). Pielou's evenness index was used to examine species evenness (53). Categorical variables were used in subgroup analysis (alpha diversity, abundance testing with LEfSe) (54) (detailed information on statistical methods can be found in Supplementary Table 2).

## Results

Study participants from different ethnic groups and diverse cultural backgrounds together provided 508 stool and 508 blood samples during the assessment period. To control for sampling bias, we restricted our microbial and statistical analysis of volatility to a subset of the cohort that had sequence data from quarterly time points with matched FC concentration which yielded 188 samples from 50 IBD patients (an additional 5 samples were removed from calculation at the sub-sampling level after sequencing and filtering as they did not have a minimum of 10,000 sequences) (Table 2).

### Baseline Assessment

**Figure 1:**
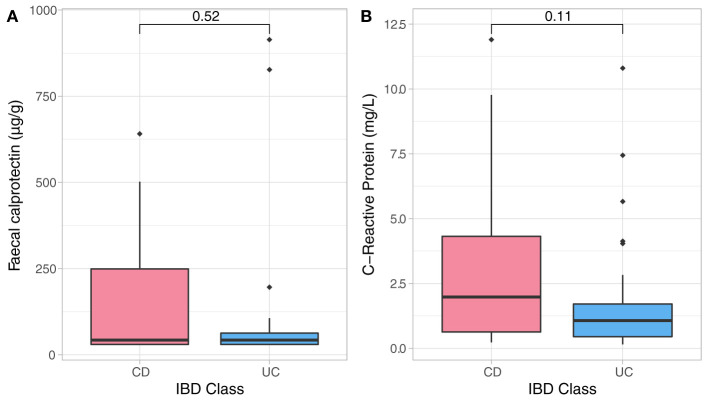
Non-parametric analysis quantifying F-calprotectin **(A)** and C-reactive protein (CRP) **(B)** at baseline and across diagnosis. Analysis did not show significant differences in measures of both CRP and FC in CD and UC participants at baseline assessment.

The entry disease remission data related to bio-psychological state of all IBD patients were employed as a benchmark for assessment of their longitudinal dynamics. Sample analysis included 50 stool and 50 blood samples from CD (*n* = 26) and UC (*n* = 24) individuals who were in clinical remission. As expected, CD and UC participants had similar baseline distributions of inflammatory biomarkers (Figure 1) which were strongly and positively associated with one another, but none were significantly related to any of the psychological measures and sleep quality at study entry. There was an analogous baseline distribution for most psychological factors, while there were significant differences in baseline scores related to depression (DASS, Dep, *p* = 0.026) and negative affectivity (DS-NA, *p* = 0.001) between CD and UC patients at reference point with UC patients showing higher baseline depression and negative affectivity scores compared to CD patients (Supplementary Table 2). Baseline anxiety scores were significantly associated with sleep disturbances (*p* < 0.001, *r*^2^ = 0.52) and baseline stress scores were strongly related to sleep duration (*p* < 0.001, *r*^2^ = 0.46). Both outcomes suggest strong relationships between anxiety and stress, and sleep — Supplementary Table 2.

### Treatment Modalities at Baseline

Both biologic and non-biologic treatment groups were similar in most baseline psychological scores with the exception of higher baseline stress scores (*p* = 0.004) and negative affectivity scores (*p* = 0.004) in non-biological group. At baseline, UC patients had higher mean psychological scores compared to CD patients in both treatment groups (except for DS-SI score which was higher in CD group who received biological treatment). These findings overall were not surprising given the mandated clinical remission at baseline—Supplementary Table 2.

### Baseline Microbiome Assessment

**Figure 2:**
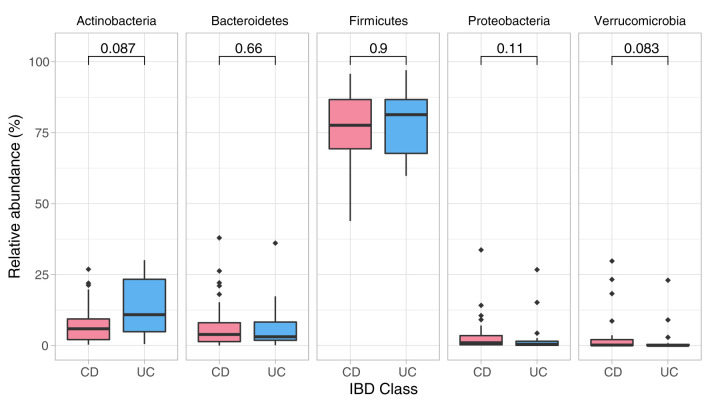
Phylum composition in baseline analysis (CD, UC). Phylum composition relative abundance for baseline analysis in all CD, UC participants. Only the top 5 phyla are shown. Numbers indicate the *p*-value from the Wilcoxon tests.

Data showed no significant baseline differences in the Shannon index (*p* = 0.67), Pielou's evenness (*p* = 0.80) and Chao1 (*p* = 0.429) between CD and UC patients. Comparison of the baseline phyla abundance is shown in Figure 2. Results showed a strong negative baseline relationship between sleep latency and Shannon index (*p* = 0.001, *r*^2^ = −0.425), Pielou's evenness (*p* = 0.002, *r*^2^ = −0.401) and Chao1 index (*p* < 0.001, *r*^2^ = −0.455), indicating that lower intestinal microbial diversity, richness, and uneven microbial composition was associated with longer time to fall asleep.

Detailed comparison of the baseline phyla abundance showed that *Verrucomicrobia* was present in 23/25 CD participants (4.0% ± 8.2), but only in 17/24 UC participants (2.3% ± 5.8). CD participants had more baseline abundance of some disease associated bacteria when compared to UC including *Bacteroidetes* (CD = 8.1% ± 9.8, UC = 6.2% ± 7.9) and *Proteobacteria* (CD = 4.2% ± 7.4, UC = 2.6% ± 6.2), but UC participants had more *Firmicutes* compared to CD at baseline assessment (CD = 75.7% ± 13.5, UC = 77.4% ± 11.8). Notwithstanding these data, none of the baseline differences were statistically significant between the CD and UC (Wilcoxon test, Figure 2; Table 3).

**Table 3:** Grouped summary IBD class: detailed comparison of the baseline phyla abundance in CD and UC participants.

**IBD_ Class**	**Phylum**	**Mean**	**SD**	***n***
CD	*Actinobacteria*	8.01	7.72	25
CD	*Bacteroidetes*	8.10	9.81	25
CD	*Firmicutes*	75.7	13.5	25
CD	*Proteobacteria*	4.22	7.35	24
CD	*Verrucomicrobia*	4.03	8.23	23
UC	*Actinobacteria*	12.3	9.59	24
UC	*Bacteroidetes*	6.24	7.92	24
UC	*Firmicutes*	77.4	11.8	24
UC	*Proteobacteria*	2.55	6.15	23
UC	*Verrucomicrobia*	2.25	5.78	17

*Verrucomicrobia is absent in 2 CDs and 7 UCs*.

### Longitudinal Assessment

We used cluster analysis to study the bio-psychological behavior of IBD participants over time. Here clusters were identified based on systematic relationships found in psycho-biological and microbiome dynamics over time and across all study participants. Three stable clusters were identified which persisted in their baseline group categories during follow-up. One potential explanation is most participants remained in clinical remission during the 12 months follow up period (Table 4A). The first cluster (No 1) was the youngest IBD cluster which earned the highest anxiety, depression, and stress scores over time and revealed worst sleep quality sores, higher measures related to depression in medically ill state during follow up (Supplementary Tables 3, 4). This cluster also had lowest number of IBD patients on biological therapy. Second cluster (No. 2) is the oldest cluster with higher number of participants in this group (both male and female and mostly UC, Table 4B—detailed information on cluster analysis is included in Supplementary Tables 20, 21).

**Table 4:** Cluster membership based on similarities found within IBD participants.

**Number of clusters**			**Freq. of IBD patients in each cluster**
**(A) Based on their baseline psychological state**.		
1			9
2			22
3			18
**Clusters**			**Total**
**(B) Based on gender, IBD disease phenotype and treatment modalities**			
**for consistency**			
Gender	F	M	
1	5	4	9
2	9	13	22
3	7	11	18
IBD phenotype	UC	CD	
1	3	6	9
2	13	9	22
3	9	9	18
Treatment modes	On-biologic	Non-biologic	
1	8	1	9
2	12	10	22
3	15	3	18

### Cluster Memberships

**Figure 3:**
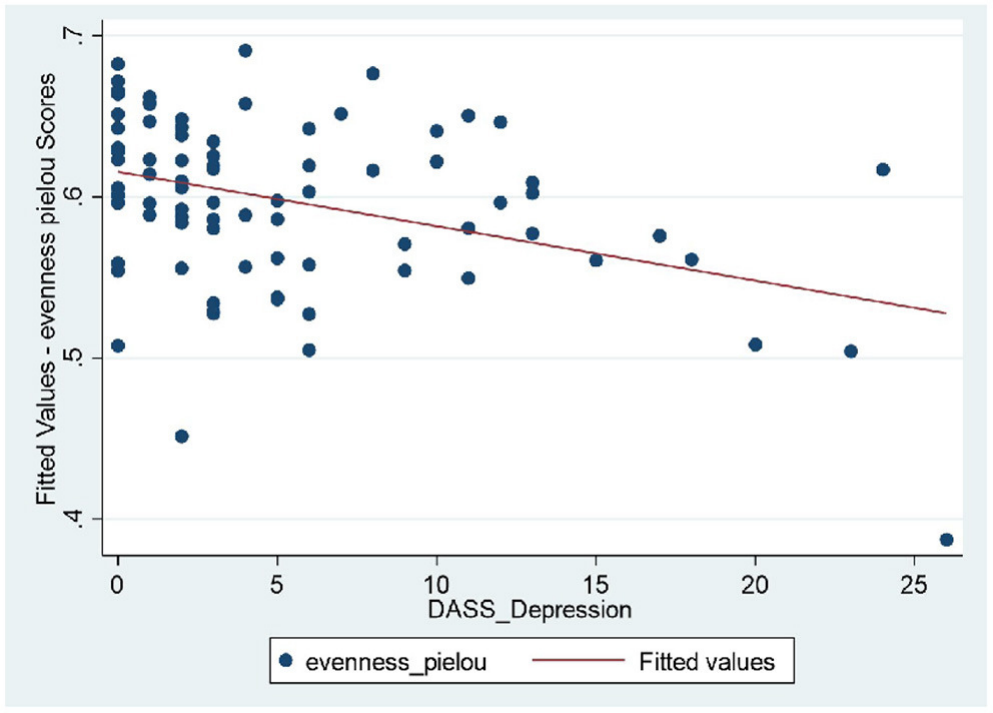
UC patients: strong negative relationship between microbial evenness and depression scores (*p* < 0.001), meaning that lower microbial evenness was strongly associated with higher depression scores in UC cohort.

Longitudinal bio-psychological data of all remission IBD patients (CD, *n* = 25; UC, *n* = 17) were analyzed (460 blood and 460 stool samples) using mixed models. Results did not show significant shift in measures of inflammatory biomarkers within IBD cohort which was expected due to persisting remission. Results identified significant longitudinal coefficient of change in psychological scores (linear, quadratic or both; Supplementary Table 2) including negative affectivity showing greater magnitude of change in the remission UC group and sleep quality showing larger magnitude of change in the remission CD group. Microbial diversity and richness displayed larger magnitude of linear coefficient of change in the remission UC group. Further examination of complete study cohort including independent variables (psychobiological factors) with outcome variables (wellbeing scores and inflammatory biomarkers) was applied. In CD patients, results suggested a statistically significant negative relationship between wellbeing scores with depressive scores (*p* = 0.018) and positive relation with sleep quality (*p* = 0.027) over time. Outcomes did not suggest any significant interdependence between longitudinal psychological scores, sleep, mode of treatment and microbial indices in CD patients. In UC patients, longitudinal wellbeing scores retained a significant and positive relationship with sleep quality (*p* = 0.040), and a significant and unexpectedly negative relationship with stress (DASS stress, *p* = 0.009). FC had a positive and significant association with sleep quality (PSQI, *p* = 0.006) and a strongly negative relationship with stress (DASS stress, *p* = 0.023) (Supplementary Table 17). Assessment of longitudinal psycho-microbial dynamics in UC cohort did not suggest any significant interdependence between longitudinal psychological scores, sleep, and mode of treatment with microbial dynamics in UC patients except for a strong negative association between microbial diversity and depression (DASS depression, *p* = 0.011), and a negative association between microbial evenness and depression (*p* < 0.001, Figure 3) but not with microbial richness, Supplementary Table 19.

### Longitudinal Microbiome Assessment

**Figure 4:**
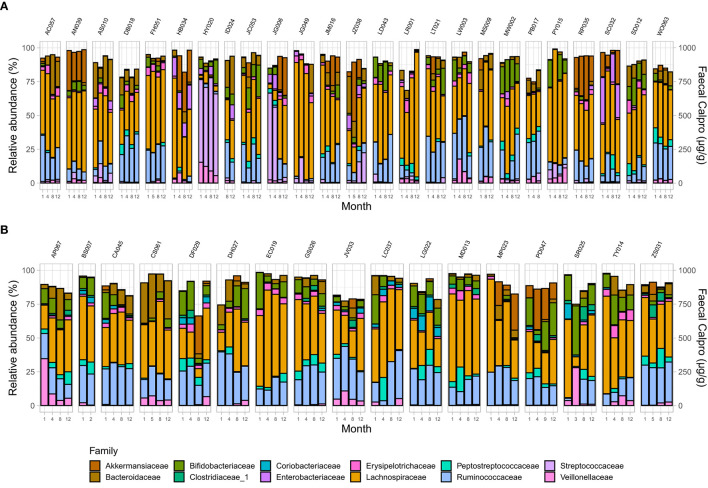
Microbial community composition at family level in patients in remission. **(A)** CD, and **(B)** UC. Only families with a relative abundance ≥2% in any sample are shown.

**Figure 5:**
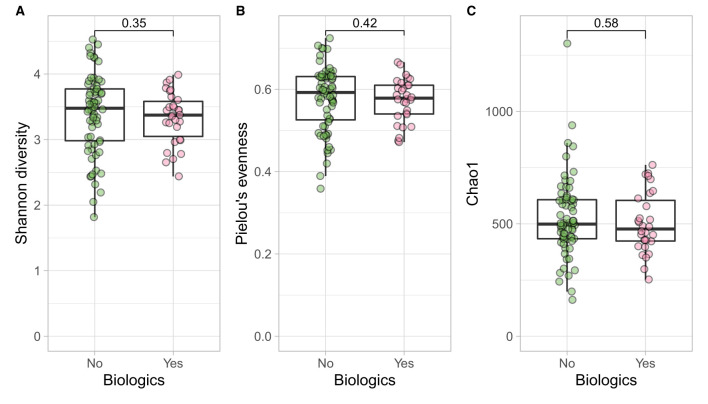
CD participants- microbial α diversity and treatment modalities. Measures of microbial α diversity between treatment options in CD group (Comprising remission and relapse) including: **(A)** Shannon's index (diversity); **(B)** Pielou's evenness; and **(C)** Chao1 index (richness). Analysis did not show any significant differences between the two groups over time.

Analysis demonstrated a significant interplay between wellbeing scores with Shannon index and with Pielou's evenness but not with Chao1 (Supplementary Table 7). To examine microbial composition shift over time, microbial dynamics were explored in remission samples of IBD subtypes in addition to changes in FC concentration longitudinally. CD cohort in clinical remission showed greater microbiome fluctuations mainly by trading off different microbial families as *well* as in relative abundance of existing microbial profile (Figures 4, 5).

### Treatment Options

Longitudinal trends of psycho-biological factors and their interactions with modes of treatment (biological vs. no biological) were examined using mixed models of analysis. In CD and UC cohorts such factors were similarly distributed in both treatment groups over time (Supplementary Table 2). At the baseline there were similar ecological indices between the two treatment options in both disease classes which was also suggested by previous study (55) (Shannon, *p* = 0.380; Pielou's, *p* = 0.246; Chao1, *p* = 0.934), although the age effect was significant between the two groups for all three ecological indices (56) (information related to sample demographics for microbial analysis and output of PERMANOVA for both biologics and bio-flare variables in CD and UC cohorts are in Supplementary Table 9).

**Figure 6:**
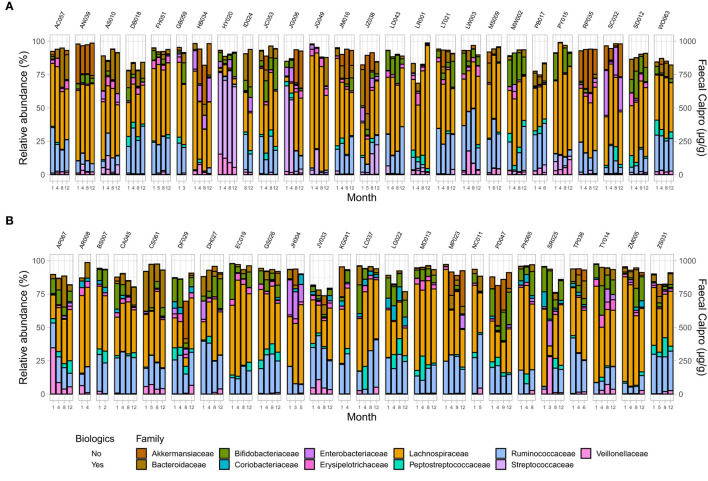
Microbial community composition at family level in IBD patients across two treatment options including changes in FC concentration over time in **(A)** CD, and **(B)** UC. Only families with a relative abundance ≥2% in any sample are shown.

**Figure 7:**
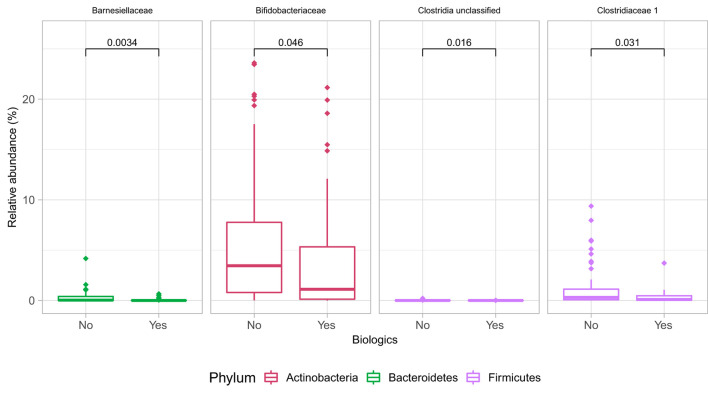
Differences in some microbial family abundances between the two treatment options in CD group (biologics vs. non-biologic). Only significantly different families are shown (Wilcoxon, *p* ≤ 0.05).

**Figure 8:**
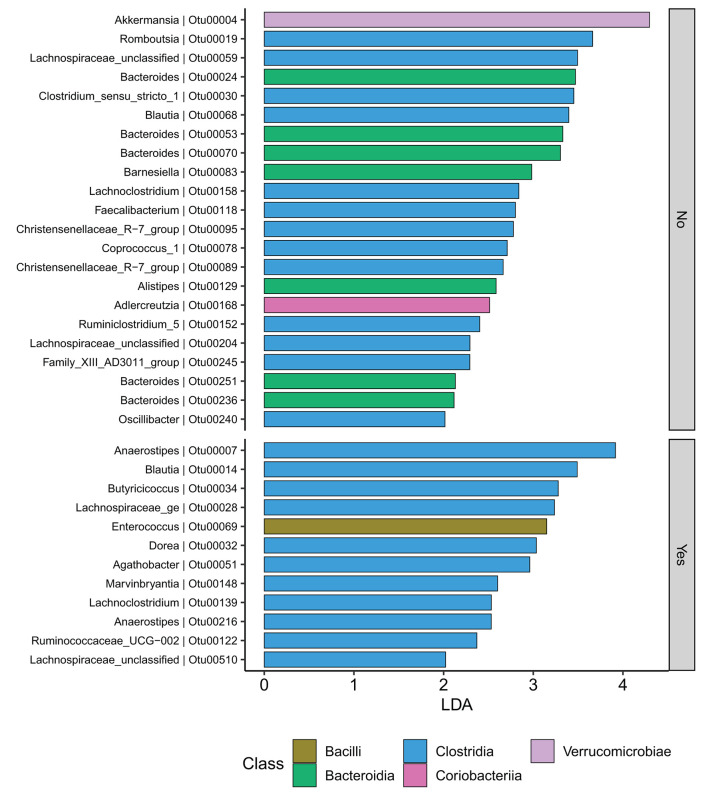
CD participants-Microbial biomarkers. OTUs microbial biomarkers for CD groups with two treatment options including biologic treatment (Yes) and non-biologic treatments (No) (OTUs and Genus information on Supplementary Table 14).

At the family level (Figure 6A), the CD group showed significant differences (Wilcoxon, *p* ≤ 0.05) between the treatment received (biologics vs. non-biologic) in the abundance of *Barnesiellaceae, Bifidobacteriaceae*, unclassified *Clostridia*, and *Clostridiaceae* (Figure 7). Results showed similar ecological index values in samples from the two treatment options. Linear discriminant analysis (LDA) with LEfSe was performed to characterize the differences between two treatment options in CD group (both in remission and during relapse). A total of 12 microbial biomarkers (OTUs) characteristic of CD under biological treatment, and 22 in CD with non-biological treatments, were identified (Figure 8).

**Figure 9:**
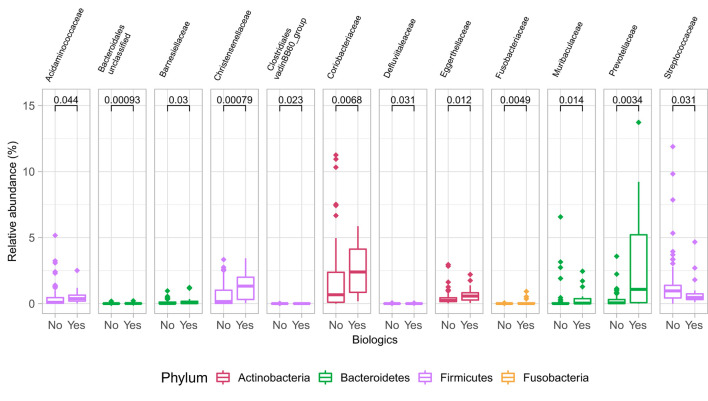
Differences in some microbial family abundances between the two treatment options in UC group. Only significant results are shown (Wilcoxon, *p* ≤ 0.05).

**Figure 10:**
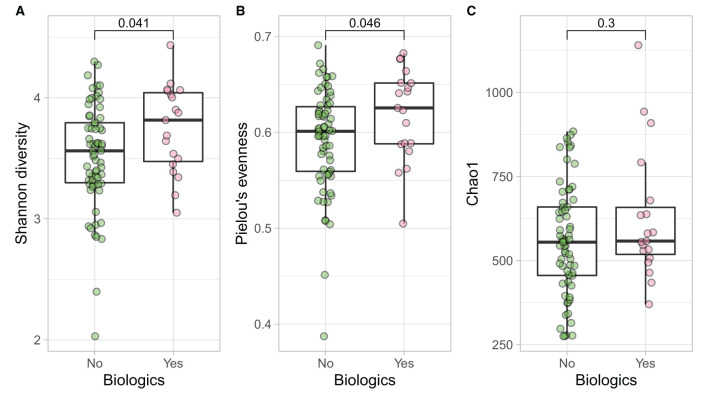
UC participants- microbial a diversity and treatment modalities. Measures of microbial alpha diversity between treatment options in UC group (comprising remission and relapse) including: **(A)** Shannon's index (diversity); **(B)** Pielou's evenness; and **(C)** Chao1 index (richness).

**Figure 11:**
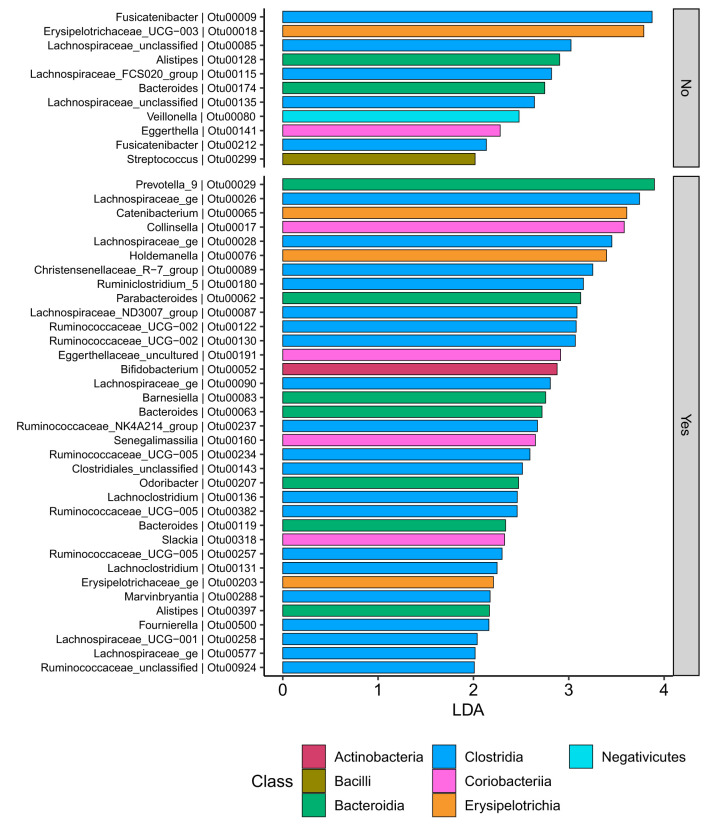
UC participants-Microbial biomarkers. OTUs microbial biomarkers for UC groups with two treatment options (biologic as Yes and non-biologic treatments as No) (OTUs and Genus information on Supplementary Information No. 14).

Samples in UC groups on biological and non-biologic treatment modes (Figure 6B) were found to have significantly different microbial communities based on Bray-Curtis dissimilarity (PERMANOVA, *p* = 0.0031). Microbial family composition in UC group and on two treatment modalities showed a total of 12 families that were significantly different (Wilcoxon, *p* ≤ 0.05): *Acidaminococcaceae*, unclassified *Bacteroidales, Barnesiellaceae, Christensenellaceae, Clostridiales* vadinBB60 group, *Coriobacteriaceae, Defluvitaleaceae, Eggerthelaceae, Fusobacteriaceae, Muribaculaceae, Prevotellaceae*, and *Streptococcaceae* (Figure 9). Results identified significant differences in microbial diversity (Shannon index, *p* = 0.041), and evenness (Pielou's evenness, *p* = 0.045) between the two treatment modes (Figure 10) (see Supplementary Table 22 for microbial dynamics across the three clusters). No differences were identified in richness (Chao1) in UC participants (Figure 10) (57). LDA of UC group between two treatment options identified 11 microbial biomarkers (OTUs) which were more abundant in UC group with no-biological treatments and 35 microbial biomarkers were identified in UC group under biological treatments (Figure 11).

## Discussion

Inflammatory Bowel Diseases are chronic and complex gut inflammatory condition, both associated with significant morbidity. IBD is correlated with a highly relevant and significant psychosocial burden (58). Early studies suggested that both CD and UC are associated with high incidence of psychological manifestation (59). A large Canadian population-based study reported 3 times higher depressive rates in IBD patients compared to healthy population, with significant higher scores during active phase of the disease (60). Greater anxiety and stress scores have been reported in patients with more sever IBD symptoms and lower compliance with treatment (61). Nevertheless, similar studies have concluded that significant number of CD patients present with depressive or anxiety symptoms, despite clinical remission, can therefore benefit from psychological support (62).

It is widely accepted that DSS-induced colitis in mice results in anxiety-like behavior that increases with and can be controlled by managing the inflammation. The degree of the DDS-induced inflammation can also be regulated by manipulation of the gut microbiota prior to DDS initiation (i.e., administration of pre and/or probiotics), which consequently prevents the behavioral deficits provoked by DSS application (63–66).

Gut microbiota dysbiosis is considered as a novel factor in the pathogenesis of IBD. Gut microbiome and its products foster a distinct effect on host immune system and promote intestinal homeostasis and healthy state. Once the symbiotic interplay between gut microbiome and its profile is disturbed, its various physiological functions will then be impaired (67–69). Yet the role and dynamics of gut microbiota in IBD development and whether the gut microbiota alteration is the cause of the intestinal inflammation or simply a product of the IBD, is not clear (70, 71).

Four major bacterial phyla: *Bacteroidetes, Firmicutes, Actinobacteria* and *Proteobacteria*, constitute more than 90% of healthy human gut bacterial species (72–74) with substantial inter-individual microbial diversity within these major phylotypes (75). In IBD patients, the dysbiosis was mostly associated with reduced bacterial diversity (predominantly in *Firmicutes* and *Bacteroidetes*) and increased bacterial species belonging to *Enterobacteriaceae* (76–78). More in-depth studies have shown a clear reduction in *Firmicutes* and significant decrease of many other beneficial bacterial species from the genera *Lactobacillus, Eubacterium* and *Bacteriodes* (79–82). Other literature addressed the association between relative abundance of a specific gut microbiome species with the mode of treatment (83–86). Such studies also examined the gut microbiome profile before and after therapeutic intervention and the duration to relapse after withdrawal. Examples would be the reduction of *Proteobacteria* in CD patients with anti TNF-α therapy (86) and increase of the abundance of *Faecalibacterium prausnitzii* in responders during the induction of anti-TNF-α antibody therapy. Another example would be the inhibitory effect of thiopurines on growth of *Mycobacterium avium* subspecies *paratuberculosis in vitro* (84). Studies also shown the correlation between reduced *Firmicutes* abundance and shorter time to relapse after Infliximab withdrawal in pediatric IBD (86).

Thus, gut microbiota may be used as a potential biomarker in respond to the treatment of IBD or can be employed to modify the host's environment and enhance the intestinal dysbiosis. Examples of the latter could be the complementary and alternative medicine (CAM), including pre and probiotics, antibiotics, fecal microbiota transplant (FMT) and nutraceuticals (87, 88).

This study was designed to characterize the longitudinal temporal trajectories of biological and psychological factors and their interdependence with disease activity and symptom manifestations in IBD patients. Assessment was made during clinical remission and at the time of relapse. At baseline analysis, all IBD patients were in clinical remission, therefore similar bio-psychological and microbiome dynamics across all IBD patients and between the disease phenotypes, were not surprising.

Results suggested a higher anxiety level was associated with greater sleep disturbances and increased stress was correlated significantly with longer sleep time in IBD participants at baseline assessment. This outcome is not surprising considering the state of clinical remission at baseline and the evidence suggested by clinical studies on the negative impact by anxiety (89) and depression (90) on sleep quality in general population. Remission CD and UC patients demonstrated significant differences in their baseline depression and negative affectivity of personality type, which entails further investigation.

Assessment of baseline microbial profile indicated that lower intestinal microbial diversity and richness, and uneven microbial composition were associated with longer time to fall asleep in both CD and UC patients. Microbial phyla abundance analysis showed that CD patients had higher abundance of some disease associated bacteria (including *Bacteroidetes* and *Proteobacteria*) compared to UC at baseline. Like previous studies (55), the baseline microbial state was similar between the two treatment modalities in CD and UC cohorts.

Longitudinal assessment of psychological factors in IBD patients who maintained clinical remission revealed strong coefficient of change although the magnitude of this longitudinal shift was not similar between remissive CD and UC patients over time. In the remission CD group, greater microbial fluctuations at the family level were observed as well as alteration in relative abundance of existing microbial profile compared to remissive UC patients over time. This outcome was also suggested by previous studies (55, 91) and might be explained by fundamental differences in the nature of the two disease phenotypes. CD patients who received biological therapy revealed similar psychobiological state to those who received non-biological treatments although there was either marginal or significant interaction between biological therapies and longitudinal state of depression and stress in this group. Comparable outcomes were detected in UC patients over time, but the significant interaction constituted by biological therapy was mainly registered on measures of inflammatory biomarkers and quality of life of these patients. The nature and mechanism of such interactions was not investigated in this study but could imply that biological therapy and their immune-physiological pathways might have play a role in sustaining the biopsychological interplay.

Longitudinal analysis on mode of treatment and gut microbiome did not identify significant differences in microbial profile in two CD treatment groups, whereas in UC group, there were significant differences in microbial diversity and evenness between the biologic and non-biologic interventions, with UC patients on biologics benefiting from more diverse and more even bacterial dynamics, but no richness differences were identified between the two. This outcome suggests that although majority of bio-psychological factors remained relentless, biological therapies potentially influenced such factors while maintaining clinical remission and might be the product of effective therapy choice in controlling the disease activity. Conceivably, this result could be related to the mechanism of action enforced by biological therapies in controlling the disease activities/maintaining remission or microbial function in responding to the therapy and requires further investigations and inclusion of multiomic analyses as well as assessment of microbial function. The differences might also reflect distinctions between clinical and deeper (e.g., endoscopic) remission, as the latter was not routinely assessed in this patient cohort. This study reinforced that lower ecological indices are significantly correlated with depression in UC patients, a feature previously identified (92, 93) and higher disease activity measures were negatively related to quality of life and sleep quality of both CD and UC patients, as previously shown (94).

### Strengths and Limitations of the Current Study and Future Directions

This study was the first prospective longitudinal study designed to evaluate bio-psychological interdependence, their complex orchestrated interplay, and their influence on the clinical course of IBDs including the treatment modalities. Time series analysis was used to examine bidirectional and longitudinal interplay between multiple disease contributing factors to measure the rebound effect. A limitation of this study was lack of healthy controls. Diet is a very significant factor affecting gut health and microbial profile (95, 96), but dietary intake was not assessed in this study. None of the participants were exmained for potential pre-existing psychological conditions. Clearly, replication of the current study is needed to further test the conceptual framework with a larger sample size, a better sample representation and by including a large cohort of healthy controls.

## Conclusion

This study indicates temporal and close interplay between psychological, immune system and microbiome dynamics in IBD patients. The mechanism of such interactions and directional sequence of such interplay remained unexplained and rather speculative, therefore requiring further investigations.

## Data Availability Statement

Raw amplicon data is available at ENA under project PRJEB43193 (https://www.ebi.ac.uk/ena/browser/view/PRJEB43193).

## Ethics Statement

This study was approved by the Human Research Ethics Committee of South Eastern Sydney Local Health District (Ref: 15/094 HREC/15/POWH 245−20 Aug). Site Specific Approval was obtained for St. George Hospital (SSA ref: 15/G/150 – 31 Aug 2015) and for St. Vincent's Hospital (Ref: 15/207 – 2 Nov 2015). The patients/participants provided their written informed consent to participate in this study.

## Author Contributions

PT: conceptualization, methodology, project administration, analysis, writing—original draft, preparation, investigation, data curation and validation, resources, and formal analysis. UV-C: supervision, writing—review, and editing. DH-P: supervision, software, formal analysis, and validation. XV-C: software, analysis, and producing results related to microbiome section. MG: supervision, conceptualization, writing—review and editing, and validation. All authors contributed to the article and approved the submitted version.

## Funding

This research was partly funded by a grant from the St George and Sutherland Medical Research Foundation. XV-C acknowledges support from the New South Wales State Government RAAP scheme and the National Collaborative Research Infrastructure Strategy.

## Conflict of Interest

The authors declare that the research was conducted in the absence of any commercial or financial relationships that could be construed as a potential conflict of interest.

## Publisher's Note

All claims expressed in this article are solely those of the authors and do not necessarily represent those of their affiliated organizations, or those of the publisher, the editors and the reviewers. Any product that may be evaluated in this article, or claim that may be made by its manufacturer, is not guaranteed or endorsed by the publisher.

## Abbreviations

CD: Crohn's disease
CRP: C-reactive protein
DASS: Depression Anxiety Stress Scale
DMI: depression in medically ill
DS-NA: Type D personality scale of negative affectivity
DS-SI: Type D personality scale of social inhibition
FC: Fecal calprotectin
IBD: Inflammatory Bowel Disease
PSQI: Pittsburgh sleep quality index.
