# Supplementary material for: The Interplay Between Use of Biological Therapies, Psychological State, and the Microbiome in IBD
Source: Front Med (Lausanne). 2022 Jul 19;9:788992. doi: 10.3389/fmed.2022.788992 (PMC9345200; doi:10.3389/fmed.2022.788992)
Supplement: Supplementary file 1 [file Data_Sheet_1.doc]

# The interplay between use of biological therapies, psychological state, and the microbiome in IBD (LIMBO study)

P. Tavakoli^1*^, U. Vollmer-Conna^2^, D. Hadzi-Pavlovic^2^, X. Vázquez-Campos^3^, M.C. Grimm^1^

^1^St George and Sutherland Clinical School, ^2^School of Psychiatry, Faculty of Medicine, and ^3^School of Biotechnology and Biomolecular Sciences, Faculty of Science, University of New South Wales, Sydney, NSW Australia

Supplementary information

SI No1.

## Inclusion and Exclusion criteria

| Longitudinal course of inflammatory bowel disease |  |
| --- | --- |
| **Attachment A: Inclusion/Exclusion Criteria**  **St George clinical school and ST Vincent’s clinical school** | |

**I. INCLUSION CRITERIA**

1. **Age**. Aged 18–80.
2. **Diagnosis of UC/CD**.
   1. CD or UC previously diagnosed on the basis of standard clinical, endoscopic, and histological criteria.
3. **Currently in Complete Clinical Remission (CCR)**. CCR is defined using the Crohn’s Disease Activity Index or the Mayo Index Score (see Attachment B):
   - 1. CDAI < 150; or
     2. Mayo Index score < 4

**II. EXCLUSION CRITERIA**

1. **Medical**.
   1. IBD- Unclassified.
   2. Significant intercurrent medical illness, including cardiac, respiratory, neurological, renal, vascular diseases or cancer (excluding non-melanoma skin cancer)
   3. Active IBD as defined by CDAI or Mayo Index, or as determined by clinical assessment
   4. Corticosteroid use (oral or topical) in last 4 weeks
   5. Recent change in drug dosage (Salicylates must be stable for ≥ 4 weeks; Azathioprine, 6MP or Methotrexate or biological agents must be stable ≥ 8 weeks)
   6. Use of Cyclosporin, Tacrolimus, Mycophenolate, or investigational agents for IBD in the last 12 weeks
2. **Completion of questionnaires**.
   1. Insufficient command of English to be able to complete questionnaires.
3. **Psychophysiological testing**.
   1. Unable to undergo physiological test procedure.

## Supplementary Figures


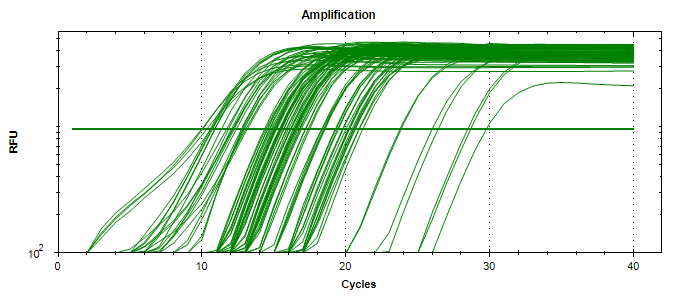
Figure S1. RT-PCR plot

## Supplementary Tables

Table S1. qPCR table

| Content | Sample | Cq | Cq Mean | Cq Std. Dev |
| --- | --- | --- | --- | --- |
| NTC |  | 29.74 | 29.74 | 0 |
| NTC |  | 28.41 | 28.41 | 0 |
| Unkn-01 |  | 15.34 | 15.38 | 0.055 |
| Unkn-01 |  | 15.41 | 15.38 | 0.055 |
| Unkn-02 |  | 14.9 | 14.98 | 0.125 |
| Unkn-02 |  | 15.07 | 14.98 | 0.125 |
| Unkn-03 |  | 18.45 | 18.37 | 0.112 |
| Unkn-03 |  | 18.29 | 18.37 | 0.112 |
| Unkn-04 |  | 15.16 | 15.3 | 0.207 |
| Unkn-04 |  | 15.45 | 15.3 | 0.207 |
| Unkn-05 |  | 12.92 | 12.81 | 0.156 |
| Unkn-05 |  | 12.7 | 12.81 | 0.156 |
| Unkn-06 |  | 23.8 | 23.76 | 0.058 |
| Unkn-06 |  | 23.72 | 23.76 | 0.058 |
| Unkn-07 |  | 25.99 | 21.58 | 6.249 |
| Unkn-07 |  | 17.16 | 21.58 | 6.249 |
| Unkn-08 |  | 14.39 | 20.37 | 8.466 |
| Unkn-08 |  | 26.36 | 20.37 | 8.466 |
| Unkn-09 |  | 10.3 | 10.14 | 0.227 |
| Unkn-09 |  | 9.98 | 10.14 | 0.227 |
| Unkn-10 |  | 12.13 | 11.9 | 0.323 |
| Unkn-10 |  | 11.67 | 11.9 | 0.323 |
| Unkn-11 |  | 16.11 | 15.97 | 0.194 |
| Unkn-11 |  | 15.84 | 15.97 | 0.194 |
| Unkn-12 |  | 19.37 | 19.39 | 0.031 |
| Unkn-12 |  | 19.42 | 19.39 | 0.031 |
| Unkn-13 |  | 10.6 | 10.32 | 0.393 |
| Unkn-13 |  | 10.04 | 10.32 | 0.393 |
| Unkn-14 |  | 12.11 | 12.08 | 0.05 |
| Unkn-14 |  | 12.04 | 12.08 | 0.05 |
| Unkn-15 |  | 16.51 | 16.44 | 0.095 |
| Unkn-15 |  | 16.38 | 16.44 | 0.095 |
| Unkn-16 |  | 20.48 | 20.52 | 0.058 |
| Unkn-16 |  | 20.57 | 20.52 | 0.058 |
| Unkn-17 |  | 12.2 | 12.27 | 0.106 |
| Unkn-17 |  | 12.34 | 12.27 | 0.106 |
| Unkn-18 |  | 14.3 | 14.39 | 0.137 |
| Unkn-18 |  | 14.49 | 14.39 | 0.137 |
| Unkn-19 |  | 15.11 | 15.23 | 0.161 |
| Unkn-19 |  | 15.34 | 15.23 | 0.161 |
| Unkn-20 |  | 16.35 | 16.32 | 0.053 |
| Unkn-20 |  | 16.28 | 16.32 | 0.053 |
| Unkn-21 |  | 17.33 | 17.37 | 0.065 |
| Unkn-21 |  | 17.42 | 17.37 | 0.065 |
| Unkn-22 |  | 18.39 | 18.44 | 0.066 |
| Unkn-22 |  | 18.48 | 18.44 | 0.066 |
| Unkn-23 |  | 28.62 | 19.57 | 12.795 |
| Unkn-23 |  | 10.52 | 19.57 | 12.795 |
| Unkn-24 |  | 14.67 | 14.85 | 0.261 |
| Unkn-24 |  | 15.04 | 14.85 | 0.261 |
| Unkn-25 |  | 16.78 | 17.53 | 1.065 |
| Unkn-25 |  | 18.28 | 17.53 | 1.065 |
| Unkn-26 |  | 20.21 | 20.33 | 0.172 |
| Unkn-26 |  | 20.45 | 20.33 | 0.172 |
| Unkn-27 |  | 10.67 | 11.24 | 0.809 |
| Unkn-27 |  | 11.82 | 11.24 | 0.809 |
| Unkn-28 |  | 15.1 | 15.25 | 0.201 |
| Unkn-28 |  | 15.39 | 15.25 | 0.201 |
| Unkn-29 |  | 17.16 | 16.83 | 0.475 |
| Unkn-29 |  | 16.49 | 16.83 | 0.475 |
| Unkn-30 |  | 20.18 | 20.39 | 0.297 |
| Unkn-30 |  | 20.6 | 20.39 | 0.297 |
| Unkn-31 |  | 10.62 | 10.96 | 0.479 |
| Unkn-31 |  | 11.3 | 10.96 | 0.479 |
| Unkn-32 |  | 14.97 | 14.88 | 0.128 |
| Unkn-32 |  | 14.79 | 14.88 | 0.128 |
| Unkn-33 |  | 16.15 | 15.89 | 0.369 |
| Unkn-33 |  | 15.63 | 15.89 | 0.369 |
| Unkn-34 |  | 20.36 | 20.04 | 0.454 |
| Unkn-34 |  | 19.72 | 20.04 | 0.454 |
| Unkn-35 |  | 10.87 | 10.87 | 0 |
| Unkn-35 |  | 10.87 | 10.87 | 0 |
| Unkn-36 |  | 14.63 | 14.56 | 0.096 |
| Unkn-36 |  | 14.49 | 14.56 | 0.096 |
| Unkn-37 |  | 16 | 16.2 | 0.279 |
| Unkn-37 |  | 16.4 | 16.2 | 0.279 |
| Unkn-38 |  | 19.77 | 19.95 | 0.258 |
| Unkn-38 |  | 20.14 | 19.95 | 0.258 |
| Unkn-39 |  | 16.15 | 15.89 | 0.373 |
| Unkn-39 |  | 15.62 | 15.89 | 0.373 |
| Unkn-40 |  | 20 | 20.46 | 0.644 |
| Unkn-40 |  | 20.91 | 20.46 | 0.644 |
| Unkn-41 |  | 16.88 | 16.66 | 0.314 |
| Unkn-41 |  | 16.44 | 16.66 | 0.314 |
| Unkn-42 |  | 15.49 | 15.67 | 0.26 |
| Unkn-42 |  | 15.85 | 15.67 | 0.26 |
| Unkn-43 |  | 19.26 | 19.24 | 0.029 |
| Unkn-43 |  | 19.22 | 19.24 | 0.029 |
| Unkn-44 |  | 16.6 | 16.66 | 0.077 |
| Unkn-44 |  | 16.71 | 16.66 | 0.077 |
| Unkn-45 |  | 21.15 | 21.02 | 0.18 |
| Unkn-45 |  | 20.89 | 21.02 | 0.18 |
| Unkn-46 |  | 12.82 | 12.79 | 0.044 |
| Unkn-46 |  | 12.76 | 12.79 | 0.044 |
| Unkn-47 |  | 13.13 | 13.08 | 0.061 |
| Unkn-47 |  | 13.04 | 13.08 | 0.061 |

| 1 | 2 | 3 | 4 | 5 | 6 | 7 | 8 | 9 | 10 | 11 | 12 |
| --- | --- | --- | --- | --- | --- | --- | --- | --- | --- | --- | --- |
| Unk1 | Unk1 | Unk9 | Unk9 | Unk17 | Unk17 | Unk25 | Unk25 | Unk33 | Unk33 | Unk41 | Unk41 |
| B |  |  |  |  |  |  |  |  |  |  |  |
| Unk2 | Unk2 | Unk10 | Unk10 | Unk18 | Unk18 | Unk26 | Unk26 | Unk34 | Unk34 | Unk42 | Unk42 |
| C |  |  |  |  |  |  |  |  |  |  |  |
| Unk3 | Unk3 | Unk11 | Unk11 | Unk19 | Unk19 | Unk27 | Unk27 | Unk35 | Unk35 | Unk43 | Unk43 |
| D |  |  |  |  |  |  |  |  |  |  |  |
| Unk4 | Unk4 | Unk12 | Unk12 | Unk20 | Unk20 | Unk28 | Unk28 | Unk36 | Unk36 | Unk44 | Unk44 |
| E |  |  |  |  |  |  |  |  |  |  |  |
| Unk5 | Unk5 | Unk13 | Unk13 | Unk21 | Unk21 | Unk29 | Unk29 | Unk37 | Unk37 | Unk45 | Unk45 |
| F |  |  |  |  |  |  |  |  |  |  |  |
| Unk6 | Unk6 | Unk14 | Unk14 | Unk22 | Unk22 | Unk30 | Unk30 | Unk38 | Unk38 | Unk46 | Unk46 |
| G |  |  |  |  |  |  |  |  |  |  |  |
| Unk7 | Unk7 | Unk15 | Unk15 | Unk23 | Unk23 | Unk31 | Unk31 | Unk39 | Unk39 | Unk47 | Unk47 |
| H |  |  |  |  |  |  |  |  |  |  |  |
| Unk8 | Unk8 | Unk16 | Unk16 | Unk24 | Unk24 | Unk32 | Unk32 | Unk40 | Unk40 | NTC | NTC |

Table S2. Assessment of bio-psychological factors at baseline and during follow up including test of any significant interplay between such factors in IBD patients.

| **Baseline assessment (CD and UC)** | **P-Value** |  | **Test of associations (Baseline)** | **P-Value** | **R^2^** |
| --- | --- | --- | --- | --- | --- |
| PSQ | 0.103 |  | F-calpro and CRP | 0.004* | 0.36 |
| Depression | 0.026* |  |  |  |  |
| Anxiety | 0.41 |  |  |  |  |
| Stress | 0.301 |  | Anxiety and sleep disturbances | <0.001 | 0.52 |
| DS-NA | 0.001* |  | Stress and sleep distribution | <0.001 | 0.46 |
| DS-SI | 0.45 |  |  |  |  |
| DMI | 0.087 |  |  |  |  |
|  |  |  |  |  |  |
|  |  |  |  |  |  |
| PSQI | 0.8 |  |  |  |  |
| F-calpro | 0.72 |  |  |  |  |
| CRP | 0.181 |  |  |  |  |
|  |  |  |  |  |  |
| **Baseline assessment and treatment modalities (Biologics Vs Conventional therapies- CD and UC)** | **Treatment modalities  *P*-Value** |  | **Longitudinal assessment -Clinical remission (Coefficient of change)** | **Linear** | **Quadratic** |
| PSQ | 0.060 |  |  | **P-Value** | |
| Stress | 0.004* |  | PSQ | 0.039* |  |
| Anxiety | 0.097 |  | Anxiety | 0.050 | 0.032* |
| Depression | 0.129 |  | DS-NA | <0.001* | 0.002* |
| DMI | 0.074 |  | PSQI | <0.001* | 0.048* |
|  |  |  | Shannon index | 0.001* |  |
|  |  |  | Chao1 | 0.012* |  |
| DS-SI | 0.264 |  |  |  |  |
| DS-Na | 0.004* |  |  |  |  |
| PSQI | 0.139 |  |  |  |  |
| F-calpro | 0.187 |  |  |  |  |
| CRP | 0.385 |  |  |  |  |
|  |  |  |  |  |  |
| **Influence made by biological therapy of  bio-psychological features in CD Cohort** | **P-Value** |  | **Influence made by biological therapy ofbio-psychological features in UC Cohort** | **P-Value** |  |
| Stress | 0.005* |  | F-calpro | 0.023* |  |
| Negative affectivity (DS-SI) | 0.019* |  | CRP | 0.009* |  |
| DMI | 0.017* |  |  |  |  |
| CRP | 0.039* |  |  |  |  |
|  |  |  |  |  |  |
| **longitudinal assessment and differences in   treatment modalities** | **Treatment modalities  *P*-Value** |  | **longitudinal assessment and differences in   treatment modalities** | **Treatment modalities  *P*-Value** |  |
| **CD** |  |  | **UC** |  |  |
| Perceived stress (PSQ) | 0.103 |  | Perceived stress (PSQ) | 0.551 |  |
| Depression (DASS Dep) | 0.36 |  | Depression (DASS Dep) | 0.742 |  |
| Anxiety (DASS Anx) | 0.657 |  | Anxiety (DASS Anx) | 0.604 |  |
| Stress (DASS Str) | 0.085 |  | Stress (DASS Str) | 0.401 |  |
| Depression in medically ill (DMI) | 0.174 |  | Depression in medically ill (DMI) | 0.674 |  |
| Social inhibition (DS-NA) | 0.054 |  | Social inhibition (DS-NA) | 0.463 |  |
| Negative affectivity (DS-SI) | 0.421 |  | Negative affectivity (DS-SI) | 0.39 |  |
|  |  |  |  |  |  |
|  |  |  |  |  |  |
| Sleep quality (PSQI) | 0.315 |  | Sleep quality (PSQI) | 0.924 |  |
| Microbial diversity (Shannon) | 0.77 |  | Microbial diversity (Shannon) | 0.184 |  |
| Microbial evenness (Pielou) | 0.904 |  | Microbial evenness (Pielou) | 0.161 |  |
| Microbial richness (Chao 1) | 0.512 |  | Microbial richness (Chao 1) | 0.301 |  |

Table S3. Psychological/ Biological variables and summary statistics; longitudinal means scores across three clusters and over 12 months of follow up (four quarters including baseline and final assessments).

| **Clusters** | Mean scores | | | | | | | | | |
| --- | --- | --- | --- | --- | --- | --- | --- | --- | --- | --- |
| Quarter 1 | Age | DASS Anxiety | DASS Depression | DASS Stress | Sleep quality | DMI |  |  | F-calpro | CRP |
| 1 | 33.33333 | 12.33333 | 13.22222 | 24.66667 | 8.666667 | 23.44444 |  |  | 71.55556 | 0.558889 |
| 2 | 45.59091 | 2.363636 | 1.863636 | 5.136364 | 4.272727 | 12.72727 |  |  | 165.1818 | 3.582273 |
| 3 | 42.66667 | 6.611111 | 1.863636 | 5.136364 | 4.272727 | 12.72727 |  |  | 165.1818 | 3.582273 |
| Quarter 2 |  |  |  |  |  |  |  |  |  |  |
| 1 | 33.33333 | 12.125 | 11 | 19 | 8.25 | 22.375 |  |  | 177 | 0.4875 |
| 2 | 45.59091 | 2.857143 | 2.190476 | 5.571429 | 4.285714 | 12.7619 |  |  | 172.3333 | 3.812857 |
| 3 | 42.66667 | 3.888889 | 2.666667 | 8.888889 | 7.388889 | 15.77778 |  |  | 140.2222 | 3.499444 |
| Quarter 3 |  |  |  |  |  |  |  |  |  |  |
| 1 | 33.33333 | 9.142857 | 9.714286 | 16.14286 | 7.428571 | 21 |  |  | 87 | 0.408571 |
| 2 | 45.59091 | 2.35 | 1.2 | 4.4 | 4.35 | 13.25 |  |  | 215.1 | 5.101 |
| 3 | 42.66667 | 6.470588 | 3.117647 | 10.70588 | 7.058824 | 15.41176 |  |  | 92.82353 | 2.252941 |
| Quarter 4 |  |  |  |  |  |  |  |  |  |  |
| 1 | 33.33333 | 15.28571 | 12.28571 | 19.85714 | 7.571429 | 24.85714 |  |  | 99.42857 | 0.495714 |
| 2 | 45.59091 | 2.823529 | 1.470588 | 5.470588 | 3.941176 | 13.58824 |  |  | 165.4706 | 3.821176 |
| 3 | 42.66667 | 4.4375 | 2.6875 | 0.3125 | 7 | 14.5625 |  |  | 136.5 | 2.086875 |

Table S4. Summary statistics (minimum, mean, maximum) of microbial dynamics including microbial diversity (Shannon), Microbial richness (Chao 1) and microbial evenness (Pielou) at the end of follow up in three clusters.

**Microbiome Variables**

| **Clusters** | Mean scores | | | |
| --- | --- | --- | --- | --- |
| Quarter 1 | Age | Microbial diversity (Shannon) | Microbial Richness (Chao 1) | Microbial Evenness (Pielou) |
| 1 | 33.33333 | 3.398545 | 532.0478 | 0.5738796 |
| 2 | 45.59091 | 3.477648 | 555.4651 | 0.5858859 |
| 3 | 42.66667 | 3.258379 | 470.4241 | 0.5638232 |
| Quarter2 |  |  |  |  |
| 1 | 33.33333 | 3.334211 | 478.8701 | 0.5764751 |
| 2 | 45.59091 | 3.462894 | 547.8643 | 0.5856079 |
| 3 | 42.66667 | 3.420438 | 541.0221 | 0.5830366 |
| Quarter3 |  |  |  |  |
| 1 | 33.33333 | 3.55026 | 544.0034 | 0.6030187 |
| 2 | 45.59091 | 3.597381 | 614.4288 | 0.5983806 |
| 3 | 42.66667 | 3.372988 | 493.3916 | 0.5779435 |
| Quarter4 |  |  |  |  |
| 1 | 33.33333 | 3.666032 | 573.7754 | 0.6185263 |
| 2 | 45.59091 | 3.491474 | 530.7913 | 0.5888444 |
| 3 | 42.66667 | 3.528833 | 580.9505 | 0.5916237 |

Table S7. Interplay between microbial profile and wellbeing scores in remission IBD. Wellbeing scores had significant effect on themselves and on measures related to microbial diversity (Shannon index), evenness (Pielou’s), but not with microbial dominance and richness (Chao1 index). Examination also did not reveal any significant impact by longitudinal measures related to microbiome dominance and richness on wellbeing score whereas diversity (Shannon) and evenness (Pielou’s) had significant impact on themselves and on wellbeing scores over the time.

|  | R² | | P |
| --- | --- | --- | --- |
|  | Wellbeing score | Chao1 |  |
| Wellbeing score |  | 0.399 | 0.066 |
| Chao1 | 0.335 |  | 0.088 |
|  |  |  |  |
|  | Wellbeing score | Microbial dominance |  |
| Wellbeing score |  | 0.306 | 0.122 |
| Microbial dominance | 0.555 |  | 0.058 |
|  |  |  |  |
|  | Wellbeing score | Microbial evenness |  |
| Wellbeing score |  | 0.317 | 0.048* |
| Microbial evenness | 0.515 |  | 0.028* |
|  |  |  |  |
|  | Wellbeing score | Microbial diversity  (Shannon) |  |
| Wellbeing score |  | 0.317 | 0.042* |
| Microbial diversity  (Shannon) | 0.551 |  | 0.032* |

Table S9. Sample frequencies in CD groups including number of samples in non-biologic CD patients, and number of samples in CD patients who received biologic treatment. Also, sample groups were included remission CD samples, pre-flare CD samples and samples from the time of relapse in CD patient who relapsed.

| Stool samples -CD | | | | |
| --- | --- | --- | --- | --- |
| Samples from  each treatment modalities |  | Frequency | Valid Percent | Cumulative Percent |
|  | Non-biologic therapy | 68 | 69.4 | 69.4 |
|  | Biologic therapy | 30 | 30.6 | 100 |
|  |  |  |  |  |
|  |  | Frequency | Valid Percent | Cumulative Percent |
| Samples categories | Remission samples | 96 | 98 | 98 |
|  | Pre-flare samples | 1 | 1 | 99 |
|  | Relapse samples | 1 | 1 | 100 |

Table S10. CD participants- Output of PERMANOVA biologics and bio-flare variables. Groups are compared based on their distances to a reference plane in a three-dimensional PCoA space based on Bray-Curtis dissimilarity distance.

| PERMANOVA | Df | Sum of Squares | R^2^ | F | Pr (>F) |
| --- | --- | --- | --- | --- | --- |
| Biologics | 1 | 0.480 | 0.01946 | 1.9055 | 0.0195* |
| Residual | 96 | 24.182 | 0.98054 |  |  |
| Total | 97 | 24.662 | 1.0000 |  |  |
| Analysis of variance | Df | Sum of Squares | Mean Square | F value | Pr (>F) |
| Groups | 1 | 0.01120 | 0.011199 | 1.0950 | 0.298 |
| Residuals | 96 | 0.98182 | 0.010227 |  |  |
| PERMANOVA | Df | Sum of Squares | R^2^ | F | Pr (>F) |
| Bio flare | 3 | 0.8351 | 0.03386 | 1.0982 | 0.2873 |
| Residual | 94 | 23.8272 | 0.96614 |  |  |
| Total | 97 | 24.6623 | 1.0000 |  |  |
| Analysis of variance | Df | Sum of Squares | Mean Square | F value | Pr (>F) |
| Groups | 3 | 0.47561 | 0.158538 | 1.0950 | 0.298 |
| Residuals | 94 | 0.96047 | 0.010227 |  |  |

Table S11. Sample frequencies in UC group including number of samples in non-biologic UC patients, and number of samples in UC patients who received biologic treatment. Also, sample groups were included remission UC samples, pre-flare UC samples and samples from the time of relapse in UC patients who relapsed.

| Stool samples -UC | | | | | |
| --- | --- | --- | --- | --- | --- |
|  |  | Frequency | Valid Percent | Cumulative Percent |  |
| Samples from each  treatment modalities | Non-biologic | 66 | 77.6 | 77.6 |  |
|  | Biologic | 19 | 22.4 | 100 |  |
|  |  | Frequency | Valid Percent | Cumulative Percent |  |
| Sample  categories | Remission | 72 | 84.7 | 84.7 |  |
|  | Pre-flared | 7 | 8.2 | 92.9 |  |
|  | Relapse | 6 | 7.1 | 100 |  |

Table S12. UC participants- Output of PERMANOVA biologics and bio-flare variables. Groups are compared based on their distances to a reference plane in a three-dimensional PCoA space based on Bray-Curtis dissimilarity distance.

| PERMANOVA | Df | Sum of Squares | R^2^ | F | Pr (>F) |
| --- | --- | --- | --- | --- | --- |
| Biologics | 1 | 0.4777 | 0.02541 | 2.1642 | 0.0031** |
| Residual | 83 | 18.3204 | 0.97459 |  |  |
| Total | 84 | 18.7981 | 1 |  |  |
| Analysis of variance | Df | Sum of Squares | Mean Square | F value | Pr (>F) |
| Groups | 1 | 0.00001 | 0.000011 | 0.0011 | 0.9733 |
| Residuals | 83 | 0.81241 | 0.009788 |  |  |
| PERMANOVA | Df | Sum of Squares | R^2^ | F | Pr (>F) |
| Bioflare | 5 | 1.5529 | 0.08261 | 1.4228 | 0.0112* |
| Residual | 79 | 17.2452 | 0.91739 |  |  |
| Total | 84 | 18.7981 | 1 |  |  |
| Analysis of variance | Df | Sum of Squares | Mean Square | F value | Pr (>F) |
| Groups | 5 | 0.11044 | 0.022088 | 2.1441 | 0.0687 |
| Residuals | 79 | 0.81384 | 0.010302 |  |  |

Table S13. Fixed-effects analysis (within) regression, **CD participants**.

Association between psychological factors (as independent variables) with wellbeing scores/inflammatory markers (as outcome variable) in CD participants over time. The overall F (3,256 df = 7.62, p< 0.0001) indicated that this model performed better than a model with no predictors. Three variables were found to be related at a statistically significant level to the well-being score and no significant associations with inflammatory biomarkers:

| **LWB_SCORE** | **Coef.** | **Std. Err** | **t** | **P>\|t\|** | **[95% Conf.** | **Interval]** |
| --- | --- | --- | --- | --- | --- | --- |
| SLEEP_Total | .0534925 | .0240082 | 2.23 | 0.027 | .0062138 | .1007713 |
| DASS_depression | -.0476939 | .0200429 | -2.38 | 0.018 | -.0871638 | -.0082241 |
| DASS_depression | -.0476939 | .0200429 | -2.38 | 0.018 | -.0871638 | -.0082241 |
| _cons | 3.607383 | .4850351 | 7.44 | 0.000 | 2.652216 | 4.56255 |
| sigma_u | .59952265 |  |  |  |  |  |
| sigma_e | .72651947 |  |  |  |  |  |
| rho | .40509912 | (fraction of variance due to u_i) |  |  |  |  |

F test that all u_i=0: F(25, 256) = 5.24 Prob > F = 0.0000

Table S14. Random-effects GLS regression: The overall chi square for the model (Wald chi (1 d.f.) = 4.67, *p* < 0.0307) indicates that this model performed better than a model with no predictors. One variable was found to be related at a statistically significant level to F CALPRO:

| **F_Calpro** | **Coef.** | **Std. Err** | **z** | **P>\|z\|** | **[95% Conf.** | **Interval]** |
| --- | --- | --- | --- | --- | --- | --- |
| DASS_Stress | -2.727967 | 1.262683 | -2.16 | 0.031 | -5.202781 | -.2531541 |
| _cons | 161.3641 | 34.49891 | 4.68 | 0.000 | 93.7475 | 228.9807 |
| sigma_u | 166.77413 |  |  |  |  |  |
| sigma_e | 71.56842 |  |  |  |  |  |
| rho | .84448344 | (fraction of variance due to u_i) |  |  |  |  |

Table S15. Random-effects GLS regression: The overall chi square for the model (Wald chi (1 d.f.) = 5.73, p < 0.0166) indicates that this model performs better than a model with no predictors. One variable was found to be related at a statistically significant level to CRP:

| **CRP** | **Coef.** | **Std. Err** | **z** | **P>\|z\|** | **[95% Conf.** | **Interval]** |
| --- | --- | --- | --- | --- | --- | --- |
| DASS_STress | -.0845224 | .0352974 | -2.39 | 0.017 | -.153704 | -.0153408 |
| _cons | 3.307481 | .6403153 | 5.17 | 0.000 | 2.052487 | 4.562476 |
| sigma_u | 2.8767974 |  |  |  |  |  |
| sigma_e | 2.1047113 |  |  |  |  |  |
| rho | .65135455 | (fraction of variance due to u_i) |  |  |  |  |

Table S16. Random-effects GLS regression: The Wald chi square for the model (Wald chi (1 d.f.) = 20.25, *p* < 0.0005) indicates that this model performs better than a model with no predictors. One variable was found to be related at a statistically significant level to CDHBI:

| **CD HBI score** | **Coef.** | **Std. Err** | **z** | **P>\|z\|** | **[95% Conf.** | **Interval]** |
| --- | --- | --- | --- | --- | --- | --- |
| _cons | 4.159194 | .4764652 | 8.73 | 0.000 | 3.225339 | 5.093048 |
| sigma_u | .48522787 |  |  |  |  |  |
| sigma_e | .54628208 |  |  |  |  |  |
| rho | .44101747 | (fraction of variance due to u_i) |  |  |  |  |

Table S17. Fixed-effects analysis (within) regression, **UC participants**.

Association between psychological factors (as independent variables) with wellbeing scores/inflammatory markers (as outcome variable) in UC participants over time. The overall chi square for the model (Wald chi (4 d.f.) = 88.20, *p* < 0.0005) indicates that this model performs better than a model with no predictors. Four variables were found to be related at a statistically significant level to the well-being score.

| **LWB_SCORE** | **Coef.** | **Std. Err** | **z** | **P>\|z\|** | **[95% Conf.** | **Interval]** |
| --- | --- | --- | --- | --- | --- | --- |
| SLEEP_Total | .0610978 | .0297238 | 2.06 | 0.040 | .0028401 | .1193554 |
| DASS_Stress | -.0336038 | .0127749 | -2.63 | 0.009 | -.0586422 | -.0085654 |
| _cons | 5.838437 | .6063754 | 9.63 | 0.000 | 4.649963 | 7.026911 |

The overall chi square for the model (Wald chi (3 d.f.) = 20.65, *p* < 0.0001) indicated that this model performed better than a model with no predictors. Three variables were found to be related at a statistically significant level to F CALPRO.

| **F_Calpro** | **Coef.** | **Std. Err** | **z** | **P>\|z\|** | **[95% Conf.** | **Interval]** |
| --- | --- | --- | --- | --- | --- | --- |
| PSQ_TOTAL | 3.577928 | 1.307815 | 2.74 | 0.006 | 1.014657 | 6.141199 |
| DASS_Stress | -5.557598 | 2.453453 | -2.27 | 0.023 | -10.36628 | -.7489176 |
| _cons | 157.4446 | 113.6201 | 1.39 | 0.166 | -65.24671 | 380.1359 |

The overall chi square for the model (Wald chi (2 d.f.) = 7.69, *p* < 0.0214) indicates that this model performs better than a model with no predictors. Two variables were found to be related at a statistically significant level to CRP.

corr(u_i, X)= 0 (assumed)Prob > chi2=0.0214

| **CRP** | **Coef.** | **Std. Err** | **z** | **P>\|z\|** | **[95% Conf.** | **Interval]** |
| --- | --- | --- | --- | --- | --- | --- |
| DMI | -.1949365 | .0850125 | -2.29 | 0.022 | -.3615578 | -.0283151 |
| _cons | 12.29592 | 3.30124 | 3.72 | 0.000 | 5.825604 | 18.76623 |
| DS14_SI | -.1200688 | .0561622 | -2.14 | 0.033 | -.2301447 | -.0099928 |
| _cons | 3.365171 | 2.196151 | 1.53 | 0.125 | -.9392053 | 7.669548 |

The Wald chi square for the model (Wald chi (1 d.f.) = 11.80, p = 0.0006) indicates that this model performs better than a model with no predictors. One variable was found to be related at a statistically significant level to MAYO binary index.

| **MAYO_bin** | **Odds Ratio** | **Std. Err** | **z** | **P>\|z\|** | **[95% Conf.** | **Interval]** |
| --- | --- | --- | --- | --- | --- | --- |
| _cons | 340.7621 | 632.1943 | 3.14 | 0.002 | 8.979887 | 12930.99 |

Table18. Random-effects, GLS regression in **CD patients** did not find any significant interplay between psychological state and microbial dynamics in full and reduced model of analysis.

| **Diversities_s~n** | **Coef.** | **Std. Err** | **z** | **P>\|z\|** | **[95% Conf.** | **Interval]** |
| --- | --- | --- | --- | --- | --- | --- |
| Sleep_Total | .0022493 | .0137017 | 0.16 | 0.870 | -.0246055 | .0291041 |
| PSQTOTAL | .0015701 | .0032911 | 0.48 | 0.633 | -.0048804 | .0080206 |
| DASS_anxiety | -.0010643 | .0109806 | -0.10 | 0.923 | -.022586 | .0204573 |
| DASS_Depression | .0063143 | .0109806 | 0.38 | 0.700 | -.0258476 | .0384761 |
| 1.bio_code | .0430011 | .1836806 | 0.23 | 0.815 | -.3170063 | .4030085 |
| Age | .0065004 | .0060299 | 1.08 | 0.281 | -.005318 | .0183187 |
| _cons | 2.9867 | .3363626 | 8.88 | 0.000 | 2.327442 | 3.645959 |
| **Evenness_pielou** | **Coef.** | **Std. Err.** | **z** | **P>\|z\|** | **[95% Conf.** | **Interval]** |
| Sleep_Total | .0015061 | .0017478 | 0.86 | 0.389 | -.0019195 | .0049318 |
| PSQTOTAL | -.0001037 | .000419 | -0.25 | 0.804 | -.0009249 | .0007175 |
| DASS_anxiety | .0001429 | .0014001 | 0.10 | 0.919 | -.0026013 | .0028871 |
| DASS_Depression | .0014341 | .002099 | 0.68 | 0.494 | -.0026798 | .0055481 |
| 1.bio_code | .0007855 | .023959 | 0.03 | 0.974 | -.0461733 | .0477443 |
| Age | .0006575 | .0007973 | 0.82 | 0.410 | -.0009052 | .0022202 |
| _cons | .5442822 | .0440264 | 12.36 | 0.000 | .457992 | .6305724 |
| **Chao1** | **Coef.** | **Std. Err.** | **z** | **P>\|z\|** | **[95% Conf.** | **Interval]** |
| Sleep_Total | -5.951508 | 5.511184 | -1.08 | 0.280 | -16.75323 | 4.850213 |
| PSQTOTAL | 1.75631 | 1.385681 | 1.27 | 0.205 | -.9595753 | 4.472196 |
| DASS_anxiety | -1.2103 | 4.47286 | -0.27 | 0.787 | -9.976944 | 7.556344 |
| DASS_Depression | -2.131178 | 6.214522 | -0.34 | 0.732 | -14.31142 | 10.04906 |
| 1.bio_code | 8.41872 | 52.92279 | 0.16 | 0.874 | -95.30804 | 112.1455 |
| Age | 2.444555 | 1.534341 | 1.59 | 0.111 | -.5626983 | 5.451807 |
| _cons | 356.154 | 102.34 | 3.48 | 0.001 | 155.5712 | 556.7368 |

Table19. Random-effects, GLS regression in **UC patients** did not find any significant interplay between psychological state and microbial dynamics -except for the negative strong relationship between microbial diversity, evenness, and depression- in full and reduced model of analysis.

| **Diversities_s~n** | **Coef.** | **Std. Err.** | **z** | **P>\|z\|** | **[95% Conf.** | **Interval]** |
| --- | --- | --- | --- | --- | --- | --- |
| Sleep_Total | .0111926 | .0170607 | 0.66 | 0.512 | -.0222458 | .044631 |
| PSQTOTAL | -.0003598 | .0047663 | -0.08 | 0.940 | -.0097015 | .0089819 |
| DASS_anxiety | .0016013 | .0087345 | 0.18 | 0.855 | -.015518 | .0187206 |
| DASS_Depression | -.0287279 | .0113544 | -2.53 | 0.011 | -.0509822 | -.0064737 |
| 1.bio_code | .1421021 | .1446978 | 0.98 | 0.326 | -.1415004 | .4257046 |
| Age | .0066351 | .0046702 | 1.42 | 0.155 | -.0025184 | .0157885 |
| _cons | 3.349175 | .38248 | 8.76 | 0.000 | 2.599528 | 4.098822 |
| **Diversities_s~n** | **Coef.** | **Std. Err.** | **z** | **P>\|z\|** | **[95% Conf.** | **Interval]** |
| DASS_Depression | -.0334864 | .0080487 | -4.16 | 0.000 | -.0492615 | -.0177113 |
| _cons | 3.730498 | .0735924 | 50.69 | 0.000 | 3.586259 | 3.874736 |
| sigma_u | .24305889 |  |  |  |  |  |
| Evenness_pielou | Coef. | Std. Err. | Z | P>\|z\| | [95% Conf. | Interval] |
| Sleep_Total | .0028337 | .002256 | 1.26 | 0.209 | -.0015879 | .0072553 |
| PSQTOTAL | -.0003237 | .0006305 | -0.51 | 0.608 | -.0015594 | .000912 |
| DASS_anxiety | .0001637 | .0011522 | 0.14 | 0.887 | -.0020946 | .002422 |
| DASS_Depression | -.0038131 | .0014988 | -2.54 | 0.011 | -.0067506 | -.0008755 |
| 1.bio_code | .0180919 | .0184429 | 0.98 | 0.327 | -.0180555 | .0542394 |
| Age | .0002835 | .0005987 | 0.47 | 0.636 | -.0008899 | .0014569 |
| _cons | .6052154 | .0499328 | 12.12 | 0.000 | .5073489 | .7030819 |
| **Chao1** | **Coef.** | **Std. Err.** | **z** | **P>\|z\|** | **[95% Conf.** | **Interval]** |
| Sleep_Total | -3.13101 | 6.832147 | -0.46 | 0.647 | -16.52177 | 10.25975 |
| PSQTOTAL | 1.14423 | 1.909669 | 0.60 | 0.549 | -2.598652 | 4.887111 |
| DASS_anxiety | -1.480013 | 3.482398 | -0.42 | 0.671 | -8.305387 | 5.345361 |
| DASS_Depression | -1.598789 | 4.532393 | -0.35 | 0.724 | -10.48212 | 7.284538 |
| 1.bio_code | 29.26891 | 54.26269 | 0.54 | 0.590 | -77.08402 | 135.6218 |
| Age | 4.491486 | 1.769652 | 2.54 | 0.011 | 1.023031 | 7.95994 |
| _cons | 334.912 | 149.6989 | 2.24 | 0.025 | 41.50753 | 628.3165 |

Table 20. Detailed information on cluster analysis.

Cluster | On-Biologic| Non-biologic | Total

| Cluster | Bio | Non-Bio | Total |
| --- | --- | --- | --- |
| 1 \| 8 1 \| 9 | | | |
| \| 88.89 11.11 \| 100.00 | | | |
| \| 22.86 7.14 \| 18.37 | | | |

| 2 \| 12 10 \| 22 |
| --- |
| \| 54.55 45.45 \| 100.00 |
| \| 34.29 71.43 \| 44.90 |

| 3 \| 15 3 \| 18 |
| --- |
| \| 83.33 16.67 \| 100.00 |
| \| 42.86 21.43 \| 36.73 |

| Total \| 35 14 \| 49 |
| --- |
| \| 71.43 28.57 \| 100.00 |
| \| 100.00 100.00 \| 100.00 |

Clusters based on the numbers of patients on biological therapy and conventional therapy in each cluster.

| **Clusters: Gender** |
| --- |
| Sex: Demographics-Gender in each cluster |
| Cluster \| F M \| Total |
|  |
| 1 \| 5 4 \| 9 |
| \| 55.56 44.44 \| 100.00 |
| \| 23.81 14.29 \| 18.37 |
|  |
| 2 \| 9 13 \| 22 |
| \| 40.91 59.09 \| 100.00 |
| \| 42.86 46.43 \| 44.90 |
|  |
| 3 \| 7 11 \| 18 |
| \| 38.89 61.11 \| 100.00 |
| \| 33.33 39.29 \| 36.73 |
|  |
| Total \| 21 28 \| 49 |
| \| 42.86 57.14 \| 100.00 |
| \| 100.00 100.00 \| 100.00 |

| **Clusters: IBD disease phenotypes** |
| --- |
| IBD: Clusters based on distribution of IBD disease phenotypes in each cluster. |
| Cluster \| CD UC \| Total |
|  |
| 1 \| 3 6 \| 9 |
| \| 33.33 66.67 \| 100.00 |
| \| 12.00 25.00 \| 18.37 |
|  |
| 2 \| 13 9 \| 22 |
| \| 59.09 40.91 \| 100.00 |
| \| 52.00 37.50 \| 44.90 |
|  |
| 3 \| 9 9 \| 18 |
| \| 50.00 50.00 \| 100.00 |
| \| 36.00 37.50 \| 36.73 |
|  |
| Total \| 25 24 \| 49 |
| \| 51.02 48.98 \| 100.00 |
| \| 100.00 100.00 \| 100.00 |

Table 21. **Psychological variables across clusters and over the time:** Psychological/ Biological variables and summary statistics (min, mean, max), longitudinal means scores across three clusters and over 12 months of follow up (four quarters including baseline and final assessments)

**Quarter 1 (Baseline)**
Summary statistics: min, mean, max
Cluster 1

| DASS_a~y | DASS_D~n | DASS_S~s | Sleep_~l | Age |  |  | F_Calpro | CRP | DMI |
| --- | --- | --- | --- | --- | --- | --- | --- | --- | --- |
| 8 | 5 | 17 | 4 | 18 |  |  | 29 | .14 | 13 |
| 12.33333 | 13.22222 | 24.66667 | 8.666667 | 33.33333 |  |  | 71.55556 | .5588889 | 23.44444 |
| 26 | 23 | 34 | 14 | 45 |  |  | 298 | 1.9 | 30 |

Cluster 2

| DASS_a~y | DASS_D~n | DASS_S~s | Sleep_~l | Age |  |  | F_Calpro | CRP | DMI |
| --- | --- | --- | --- | --- | --- | --- | --- | --- | --- |
| 0 | 0 | 0 | 1 | 20 |  |  | 29 | .14 | 10 |
| 2.363636 | 1.863636 | 5.136364 | 4.272727 | 45.59091 |  |  | 165.1818 | 11.9 | 19 |
| 11 | 6 | 12 | 8 | 76 |  |  | 827 | 11.9 | 19 |

Cluster 3

| DASS_a~y | DASS_D~n | DASS_S~s | Sleep_~l | Age |  |  | F_Calpro | CRP | DMI |
| --- | --- | --- | --- | --- | --- | --- | --- | --- | --- |
| 0 | 0 | 3 | 2 | 19 |  |  | 29 | .26 | 11 |
| 6.611111 | 3.388889 | 12.72222 | 9.277778 | 42.66667 |  |  | 105.4444 | 2.572245 | 16.16667 |
| 14 | 7 | 27 | 14 | 76 |  |  | 914 | 10.8 | 27 |

Total

| DASS_a~y | DASS_D~n | DASS_S~s | Sleep_~l | Age |  |  | F_Calpro | CRP | DMI |
| --- | --- | --- | --- | --- | --- | --- | --- | --- | --- |
| 0 | 0 | 0 | 1 | 18 |  |  | 29 | .14 | 10 |
| 5.755102 | 4.510204 | 11.5102 | 6.918367 | 42.26531 |  |  | 126.0408 | 2.572245 | 15.95918 |
| 26 | 23 | 34 | 14 | 76 |  |  | 914 | 11.9 | 30 |

**Quarter 2**

Summary statistics: min, mean, max

Cluster 1

| DASS_a~y | DASS_D~n | DASS_S~s | Sleep_~l | Age |  |  | F_Calpro | CRP | DMI |
| --- | --- | --- | --- | --- | --- | --- | --- | --- | --- |
| 0 | 0 | 3 | 3 | 18 |  |  | 29 | .14 | 13 |
| 12.125 | 11 | 19 | 8.25 | 33.33333 |  |  | 177 | .4875 | 22.375 |
| 27 | 26 | 29 | 15 | 45 |  |  | 863 | 1.09 | 29 |

Cluster 2

| DASS_a~y | DASS_D~n | DASS_S~s | Sleep_~l | Age |  |  | F_Calpro | CRP | DMI |
| --- | --- | --- | --- | --- | --- | --- | --- | --- | --- |
| 0 | 0 | 0 | 0 | 20 |  |  | 29 | .18 | 10 |
| 2.857143 | 2.190476 | 5.571429 | 4.285714 | 45.59091 |  |  | 172.3333 | 3.812857 | 12.7619 |
| 12 | 7 | 15 | 11 | 76 |  |  | 833 | 22.5 | 25 |

Cluster 3

| DASS_a~y | DASS_D~n | DASS_S~s | Sleep_~l | Age |  |  | F_Calpro | CRP | DMI |
| --- | --- | --- | --- | --- | --- | --- | --- | --- | --- |
| 0 | 0 | 0 | 2 | 19 |  |  | 29 | .14 | 10 |
| 3.888889 | 2.666667 | 8.888889 | 7.388889 | 42.66667 |  |  | 140.2222 | 3.499444 | 15.77778 |
| 14 | 11 | 25 | 12 | 76 |  |  | 776 | 11 | 30 |

Total

| DASS_a~y | DASS_D~n | DASS_S~s | Sleep_~l | Age |  |  | F_Calpro | CRP | DMI |
| --- | --- | --- | --- | --- | --- | --- | --- | --- | --- |
| 0 | 0 | 0 | 0 | 18 |  |  | 29 | .14 | 10 |
| 4.829787 | 3.87234 | 9.12766 | 6.148936 | 42.26531 |  |  | 160.8298 | 3.126809 | 15.55319 |
| 27 | 26 | 29 | 15 | 76 |  |  | 863 | 22.5 | 30 |

**Quarter 3**

Summary statistics: min, mean, max

Cluster 1

| DASS_a~y | DASS_D~n | DASS_S~s | Sleep_~l | Age |  |  | F_Calpro | CRP | DMI |
| --- | --- | --- | --- | --- | --- | --- | --- | --- | --- |
| 0 | 0 | 2 | 4 | 18 |  |  | 29 | .14 | 14 |
| 9.142857 | 9.714286 | 16.14286 | 7.428571 | 33.33333 |  |  | 87 | .4085714 | 21 |
| 31 | 18 | 29 | 12 | 45 |  |  | 197 | 1.23 | 34 |

Cluster 2

| DASS_a~y | DASS_D~n | DASS_S~s | Sleep_~l | Age |  |  | F_Calpro | CRP | DMI |
| --- | --- | --- | --- | --- | --- | --- | --- | --- | --- |
| 0 | 0 | 0 | 1 | 20 |  |  | 29 | .14 | 10 |
| 2.35 | 1.2 | 4.4 | 4.35 | 45.59091 |  |  | 215.1 | 5.101 | 13.25 |
| 10 | 4 | 14 | 10 | 76 |  |  | 773 | 17.7 | 22 |

Cluster 3

| DASS_a~y | DASS_D~n | DASS_S~s | Sleep_~l | Age |  |  | F_Calpro | CRP | DMI |
| --- | --- | --- | --- | --- | --- | --- | --- | --- | --- |
| 0 | 0 | 0 | 1 | 19 |  |  | 29 | .1 | 10 |
| 6.470588 | 3.117647 | 10.70588 | 7.058824 | 42.66667 |  |  | 92.82353 | 2.252941 | 15.41176 |
| 18 | 10 | 26 | 16 | 76 |  |  | 556 | 7.3 | 30 |

Total

| DASS_a~y | DASS_D~n | DASS_S~s | Sleep_~l | Age |  |  | F_Calpro | CRP | DMI |
| --- | --- | --- | --- | --- | --- | --- | --- | --- | --- |
| 0 | 0 | 0 | 1 | 1 |  |  | 29 | .14 | 10 |
| 5.022727 | 3.295455 | 8.704545 | 5.886364 | 42.26531 |  |  | 147.4773 | 3.254091 | 15.31818 |
| 31 | 18 | 29 | 16 | 76 |  |  | 773 | 17.7 | 34 |

**Quarter 4 (End of the follow up)**

Summary statistics: min, mean, max

Cluster 1

| DASS_a~y | DASS_D~n | DASS_S~s | Sleep_~l | Age |  |  | F_Calpro | CRP | DMI |
| --- | --- | --- | --- | --- | --- | --- | --- | --- | --- |
| 1 | 3 | 6 | 3 | 18 |  |  | 29 | .14 | 10 |
| 15.28571 | 12.28571 | 19.85714 | 7.571429 | 33.33333 |  |  | 99.42857 | .4957143 | 24.85714 |
| 38 | 24 | 28 | 11 | 45 |  |  | 310 | 1.46 | 36 |

Cluster 2

| DASS_a~y | DASS_D~n | DASS_S~s | Sleep_~l | Age |  |  | F_Calpro | CRP | DMI |
| --- | --- | --- | --- | --- | --- | --- | --- | --- | --- |
| 0 | 0 | 0 | 1 | 20 |  |  | 29 | .14 | 10 |
| 2.823529 | 1.470588 | 5.470588 | 3.941176 | 45.59091 |  |  | 165.4706 | 3.821176 | 13.58824 |
| 27 | 13 | 29 | 10 | 76 |  |  | 780 | 16.9 | 36 |

Cluster 3

| DASS_a~y | DASS_D~n | DASS_S~s | Sleep_~l | Age |  |  | F_Calpro | CRP | DMI |
| --- | --- | --- | --- | --- | --- | --- | --- | --- | --- |
| 0 | 0 | 1 | 1 | 19 |  |  | 29 | .2 | 10 |
| 4.4375 | 2.6875 | 8.3125 | 7 | 42.66667 |  |  | 136.5 | 2.086875 | 14.5625 |
| 22 | 12 | 17 | 16 | 76 |  |  | 1000 | 10.4 | 28 |

Total

| DASS_a~y | DASS_D~n | DASS_S~s | Sleep_~l | Age |  |  | F_Calpro | CRP | DMI |
| --- | --- | --- | --- | --- | --- | --- | --- | --- | --- |
| 0 | 0 | 0 | 1 | 18 |  |  | 29 | .14 | 10 |
| 5.65 | 3.85 | 9.125 | 5.8 | 42.26531 |  |  | 142.325 | 2.5455 | 15.95 |
| 38 | 24 | 29 | 16 | 76 |  |  | 1000 | 16.9 | 36 |

Table 22. **Microbiome dynamics across clusters and over the time:** Summary statistics (minimum, mean, maximum) of microbial dynamics including microbial diversity (Shannon), richness (Chao1) and evenness (Pielou’s) scores four quarters including baseline (quarter 1) and at the end of follow up (quarter 4) in three clusters.

**Quarter 1**

| Cluster \| diversity Chao1 evenness |
| --- |
|  |
| 1 \| 2.863971 395.0179 .5041233 |
| \| 3.398545 532.0478 .5738796 |
| \| 4.322996 680.971 .700218 |
|  |
| 2 \| 2.39814 252.5455 .4513545 |
| \| 3.477648 555.4651 .5858859 |
| \| 4.002765 836.7692 .6656697 |
|  |
| 3 \| 2.049106 281.7576 .3893647 |
| \| 3.258379 470.4241 .5638232 |
| \| 4.271244 873.9507 .6980458 |
|  |
| Total \| 2.049106 252.5455 .3893647 |
| \|3.382571 519.9244 .575576 |
| \|4.322996 873.9507 .700218 |

**Quarter 2**

| Cluster\| diversity Chao1 evenness |
| --- |
|  |
| 1 \|2.030115 297.3704 .3872974 |
| \|3.334211 478.8701 .5764751 |
| \|4.451366 844.7059 .699189 |
| - |
| 2 \|2.468422 277 .4596143 |
| \|3.462894 547.8643 .5856079 |
| \|4.042791 863.8993 .660309 |
|  |
| 3 \|2.435871 162.0435 .4805079 |
| \|3.420438 541.0221 .5830366 |
| \|4.277387 943.0789 .6822971 |
|  |
| Total \|2.030115 162.0435 .3872974 |
| \|3.424731 533.5002 .5830686 |
| \|4.451366 943.0789 .699189 |

**Quarter 3**

| Cluster\| diversity Chao1 evenness |
| --- |
|  |
| 1 \| 2.706542 275.0789 .4870661 |
| \| 3.55026 544.0034 .6030187 |
| \| 4.526173 735.1235 .7241906 |
|  |
| 2 \| 2.96304 350.2273 .5354837 |
| \| 3.597381 614.4288 .5983806 |
| \| 4.259303 1302.224 .665805 |
|  |
| 3 \|1.817712 199 .3586006 |
| \| 3.372988 493.3916 .5779435 |
| \| 4.194058 799.8736 .6824651 |
|  |
| Total \| 1.817712 199 .3586006 |
| \| 3.503187 556.4604 .5912223 |
| \| 4.526173 1302.224 .7241906 |

**Quarter 4**

| Cluster \| diversity Chao1 evenness |
| --- |
|  |
| 1 \| 3.291797 278 .5605809 |
| \| 3.666032 573.7754 .6185263 |
| \| 4.402512 859.791 .7061632 |
|  |
| 2 \| 2.796004 338 .4878988 |
| \| 3.491474 530.7913 .5888444 |
| \| 4.015358 698.5484 .6482233 |
|  |
| 3 \| 2.316888 269.7188 .4447441 |
| \| 3.528833 580.9505 .5916237 |
| \| 4.10067 909.0706 .6717132 |
|  |
| Total \| 2.316888 269.7188 .4447441 |
| \| 3.536965 558.3772 .5951505 |
| \| 4.402512 909.0706 .7061632 |
|  |
